# Mechanism of Ganoderma lucidum polysaccharides in the mangement of diabetes and its complications

**DOI:** 10.3389/fphar.2026.1805808

**Published:** 2026-05-01

**Authors:** Yu Xin, Xiwu Zhang, Di Han, Qichao Liang, Ling Kong, Yu Guan, Hui Sun, Chang Liu, Ying Han, Xiaoyu Wang, Xijun Wang

**Affiliations:** 1 State Key Laboratory of Integration and Innovation of Classic Formula and Modern Chinese Medicine, National Chinmedomics Research Center, National TCM Key Laboratory of Serum Pharmacochemistry, Metabolomics Laboratory, Department of Pharmaceutical Analysis, Heilongjiang University of Chinese Medicine, Harbin, China; 2 Technology Innovation Center of Wusulijiang Ciwujia, Hulin, China; 3 State Key Laboratory of Quality Research in Chinese Medicine, Macau University of Science and Technology, Taipa, Macao SAR, China

**Keywords:** combined therapy, diabetes, diabetic cardiomyopathy, diabetic nephropathy, Ganoderma lucidum polysaccharides, glycolipid metabolism, oxidative stress

## Abstract

Characterized by a total or partial shortage of insulin, diabetes is a chronic metabolic condition. Its widespread occurrence and related complications present a significant global public health issue. Ganoderma lucidum (Curtis) P. Karst. 1881 (Polyporaceae), a valuable traditional Chinese medicine, contains polysaccharides as its primary active metabolites. Polysaccharides exhibit virous pharmacological properties including hypoglycemic, hypolipidemic, immunomodulatory, and anti-tumor effects. Although recent research has increasingly investigated the potential of G. lucidum polysaccharides (GLPs) to manage diabetes and its complications, a systematic integration of these findings is lacking. The review comprehensively summarizes relevant literature retrieved from databases including PubMed, Sci-Hub, Science Direct, Scopus, and Open Access Library. GLPs demonstrates defensive impacts against diabetes and its associated complications including nephropathy, hepatopathy, cardiomyopathy, refractory wound healing, neuropathy, retinopathy, and erectile dysfunction--through mechanisms involving oxidative stress modulation, glucolipid metabolism regulation, anti-apoptosis, islet cells repair, and the gut microbiota remodeling. Furthermore, drug combination strategies and novel formulation development represent promising directions for future applications This review provides a theoretical foundation for the advancement of effective and low toxicity natural therapies for diabetes based on GLPs.

## Highlights

- This study systematically expounded the main mechanism of action of Ganoderma lucidum polysaccharides in treating diabetic complications.
- GLPs can alleviate diabetic complications by regulating the composition of intestinal flora and their metabolites, improving intestinal barrier function and inflammatory responses.
- Combined therapy and structural innovation are expected to become the key driving forces for breaking through the bottleneck in diabetes treatment.

## 1 Introduction

In the 21st century, diabetes has emerged as a critical worldwide public health threat. The 2024 edition of the International Diabetes Federation (IDF) Diabetes Atlas reports that roughly 589 million adults—equivalent to 11.1% of the global adult population—are living with diabetes, with estimates indicating this figure could rise to 853 million by the year 2050. In 2024, diabetes was responsible for over 3.4 million fatalities worldwide, while associated healthcare costs surpassed $1 trillion for the first time. The outlook is especially concerning in China. A 2025 report released by the Chinese Center for Disease Control and Prevention revealed that diabetes affects approximately 233 million individuals—15.9% of the national population—marking a surge of over 150% compared to the 2005 figure. Notably, the diabetes prevalence in adults under 40 has risen twofold, prompting national guideline revisions that lowered the recommended age for routine screening from 40 to 35 years (Duncan et al., 2025; Gao et al., 2025; Genitsaridi et al., 2026; Zhang H. et al., 2025). While first-line antihyperglycemic agents—including metformin, sulfonylureas, and exogenous insulin—achieve robust glycemic control, their chronic administration is frequently constrained by dose-dependent adverse effects (Sims et al., 2021). As shown in Table 1, these adverse reactions include gastrointestinal discomfort and the risk of lactic acidosis (for biguanide drugs), severe hypoglycemia (for sulfonylurea drugs), and possible exacerbation of insulin resistance (for insulin). These safety limitations—particularly the potential for hepatorenal toxicity, mitochondrial dysfunction, and off-target metabolic effects—have underscored the critical need for well-tolerated (Pernicova and Korbonits, 2014), organ-sparing therapeutic alternatives (Davidson et al., 2004), fueling growing scientific and clinical interest in bioactive natural compounds with favorable pharmacological and toxicological profiles (Chiefari et al., 2017; Gariboldi et al., 2023) (Table 1). The escalating disease burden underscores an urgent demand for safe, multi-mechanistic interventions—among which natural polysaccharides, backed by robust evidence of hypoglycemic efficacy, represent a promising therapeutic avenue.

**TABLE 1:** Common drugs used in clinical treatment of diabetes and their pharmacological safety characteristics.

Classify		Representative drug	Hypoglycemic mechanism	Adverse reaction	Contraindication	Relevance to natural therapies
Biguanides (first-line drugs)		Metformin	Inhibit hepatic glycogenolysis, improve insulin resistance, promote lipolysis	Gastrointestinal reactions, skin allergic reactions, lactic acidosis reactions	Patients with liver insufficiency, severe infection, hypoxia, or those undergoing major surgery	Search for more gentle alternative or supplementary treatments for the gastrointestinal tract and liver and kidneys
Insulin secretagogues	Sulfonylureas (SU)	Sulfonylureas**	Stimulate insulin secretion (secretion independent of blood glucose concentration)	Severe hypoglycemia, weight gain	T1DM, severe complications, T2DM with very poor insulin function	The flavonoids and terpenoids present in natural medicines can improve insulin resistance through various pathways, without the risk of hypoglycemia
	Glitinides	*Glitinides	Promote the early-phase secretion of insulin	Severe hypoglycemia, weight gain (less severe than SU symptoms)	Same as SU	
Thiazolidinediones		**Thiazolidinediones	Increase the sensitivity of target cells to insulin	Weight gain, edema, fracture, and an increased risk of heart failure	Heart failure, active liver disease, severe osteoporosis, history of fractures	The demand for natural products that can improve insulin resistance and have good cardiovascular safety
α-glucosidase inhibitor		Acarbose	Delay the absorption of sugar in the small intestine	Gastrointestinal reactions	Use with caution in patients with hepatic and renal insufficiency	Searching for natural medicines that are gentle on the liver and kidneys

Plant-derived polysaccharides have emerged as a focus of intensive scientific investigation in recent years due to their considerable potential as therapeutic agents for diabetes management. Clinical evidence demonstrates that Lycium barbarum polysaccharides (LBP) administration leads to a marked reduction in fasting blood glucose levels in patients with diabetes (Ma et al., 2022; Wan et al., 2022). Mulberry leaf polysaccharides protect pancreatic β-cells from apoptosis by modulating the Bax/Bcl-2 protein ratio and enhancing the expression of PDX-1—a critical transcription factor essential for insulin gene expression; they modulate insulin secretion and key components of insulin signaling pathways, thereby preserving pancreatic β-cell functionality; Moreover, they foster the expansion of beneficial gut microbes while suppressing the growth of pathogenic species—contributing to a healthier gut microbiota composition that supports hypoglycemic effects. Medicinal–food polysaccharides (MFPPs) extracted from traditionally used plants—including *Astragalus membranaceus*, *Panax ginseng*, *Pueraria lobata* root, and *Lycium barbarum*—have demonstrated efficacy in glycemic regulation and mitigation of diabetes-associated complications (He and Cui, 2025; Wu G. et al., 2024; Zhang W. et al., 2025). In addition, dietary non-starch polysaccharides (NSPs) exhibit therapeutic potential in alleviating diabetic microvascular disorders—such as nephropathy, retinopathy, and delayed wound repair—by targeting underlying pathological mechanisms (Chen L. et al., 2025; Deng et al., 2025; He et al., 2010; Li et al., 2025; Yang et al., 2025).

Natural edible fungi contain anti-diabetic active metabolites such as polysaccharides and alkaloids (Khursheed et al., 2020). Due to their natural origin, safety, strong anti-diabetic activity and multi-target treatment characteristics, they are expected to become the main focus of future diabetes treatment (Mfopa et al., 2021). Ganoderma lucidum (Curtis) P. Karst. 1881 (Polyporaceae), esteemed as a valuable botanical drug in traditional Chinese medicine, exhibits a broad spectrum of pharmacological activities (Du et al., 2024). These include antioxidant properties, immune modulation, hypoglycemic effects, anti-inflammatory actions, and anticancer potential (Kebaili et al., 2021; Li Q. et al., 2018; Shahid et al., 2023). Its main active ingredient, polysaccharides (GLPs), treats complications including nephropathy, liver injury, cardiomyopathy, neuropathy, retinopathy, and erectile dysfunction due to diabetes through multi-target pathways, including improving glucose and lipid metabolism (Jones, 2016), regulating oxidative stress (Yamada et al., 2020), inhibiting cell apoptosis (Xia et al., 2021), repairing islet cells (Li et al., 2017), alleviating inflammatory responses (Wang L. et al., 2025), and regulating the balance of the microbiota (Guo et al., 2021). Thus, it provides multi-dimensional intervention strategies for the treatment of diabetes (Zhang et al., 2012).

While prior reviews have predominantly addressed isolated biological properties of G. lucidum, a holistic and systematic evaluation of its GLPs across the full range of diabetic complications remains notably lacking. Moreover, the interconnected pathways linking gut microbiota modulation to hypoglycemic effects—and the emerging potential of novel delivery systems—have yet to be comprehensively synthesized and analyzed. Accordingly, this review seeks to provide a timely and integrative synthesis of the therapeutic potential of GLPs—not glucagon-like peptides—in managing diabetes and its multifaceted complications. We systematically evaluated plausible multi-target mechanisms, highlighted the growing importance of gut–organ crosstalk, and discussed prospective research avenues—such as structure–activity relationship studies and synergistic combination approaches—to establish a conceptual framework for advancing GLPs-derived natural therapeutics.

## 2 Methods

### 2.1 Search strategy

This review adhered to the Preferred Reporting Items for Systematic Reviews and Meta-Analyses (PRISMA) framework. A comprehensive, systematic search was performed across multiple scholarly databases—including PubMed, ScienceDirect, Scopus, the Open Access Library, and Sci-Hub—targeting publications indexed between January 2000 and January 2026. The search strategy integrated relevant keywords—covering both therapeutic interventions and disease conditions—as well as standardized Medical Subject Headings (MeSH) terms. Key search concepts included *Ganoderma lucidum* polysaccharides, diabetes mellitus, inflammatory responses, programmed cell death (apoptosis), insulin resistance and sensitivity, oxidative damage, pancreatic islet function, glycolipid homeostasis, gut microbiota modulation, multimodal or integrative therapies, structural or formulation advancements, diabetic nephropathy, hepatotoxicity, cardiovascular complications, chronic non-healing diabetic ulcers, neurodegenerative and neuropathic manifestations, and diabetic retinopathy. Only English-language publications were included in the search. Furthermore, backward citation tracking—i.e., hand-searching the reference sections of retrieved articles—was performed to uncover potentially eligible studies missed during the initial database screening.

### 2.2 Inclusion and exclusion criteria

Eligible studies satisfied all of the following criteria: (1) Primary research reports—including cell-based experiments, animal models, or human clinical trials—investigating the biological or therapeutic impact of GLPs on diabetes mellitus or glucose homeostasis; and (2) Studies that explicitly reported quantitative or qualitative outcome measures pertinent to glycemic regulation, insulin responsiveness, or the progression, prevention, or management of diabetic complications. Studies were excluded if they met any of the following conditions: (1) Non-research outputs—such as conference abstracts, narrative or systematic reviews, commentaries, or editorials—that lacked original empirical findings; (2) Investigations in which GLPs were not the primary intervention or were not explicitly evaluated for their biological or therapeutic effects; (3) Reports with insufficient methodological detail, missing outcome data, or inaccessible full-text information precluding reliable assessment.

### 2.3 Quality assessment

The SYRCLE’s Risk of Bias tool was employed to rigorously evaluate the methodological rigor of all included preclinical animal studies. For *in vitro* investigations, quality appraisal focused on the transparency and completeness of experimental procedures, as well as the consistency and replicability of reported findings.

### 2.4 Data synthesis

Due to heterogeneity in study designs and outcome measures, a narrative synthesis was conducted. The findings were organized according to the underlying mechanisms of action.

## 3 Preventive and therapeutic mechanisms of Ganoderma lucidum polysaccharides in diabetes

**FIGURE 1:**
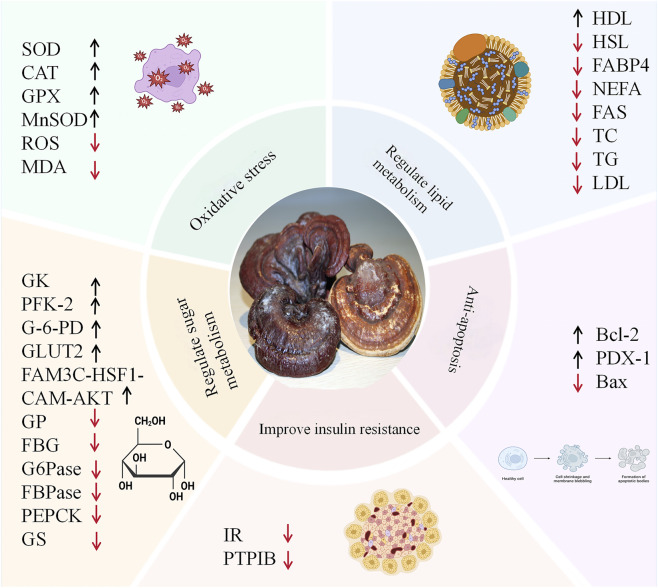
The way GLPs functions in managing diabetes.

The mechanism of GLPs in preventing and treating diabetes is rather complex. This section comprehensively elaborates on how GLPs mainly treat diabetes by regulating oxidative stress in diabetic patients, improving glycolipid metabolism disorders, inhibiting cell apoptosis, repairing pancreatic islet cells, and suppressing inflammatory responses (Ren L. et al., 2020; Zhang et al., 2022) (Figure 1).

### 3.1 Regulate oxidative stress and protect pancreatic islet cells

Reactive oxygen species (ROS) readily interact with cellular macromolecules, resulting in oxidative damage to proteins, DNA and lipids. This oxidative stress is implicated in the pathogenesis of various diseases, including diabetes, neurodegenerative disorders, aging-related conditions, and tumorigenesis (Agarwal and Sohal, 1993). Antioxidant enzymes constitute the primary defense mechanism for organisms against reactive oxygen species (ROS). Cells and organisms contain a variety of antioxidant enzymes, including superoxide dismutase (SOD), catalase (CAT), glucose-6-phosphate dehydrogenase (G6PD), glutathione S-transferase (GST), glutathione peroxidase (GPx), glutathione reductase (GR), and manganese superoxide dismutase (Mn-SOD). Reduced glutathione (GSH) is also a key antioxidant, though it is not an enzyme. These antioxidants inhibit free radical formation and facilitate the repair of oxidative damage (Chandra et al., 1994; Shi et al., 2021; YouGuo et al., 2009).

Chronic hyperglycemia triggers oxidative stress predominantly through the impairment of endogenous antioxidant systems leading to uncontrolled buildup of reactive ROS and subsequent macromolecular damage. This excessive ROS burden inflicts direct oxidative damage to mitochondrial structures and electron transport chain components in pancreatic β-cells, while concurrently disrupting key insulin signaling nodes thereby compromising glucose-stimulated insulin secretion and exacerbating peripheral insulin resistance. Critically, these impairments act synergistically to sustain elevated blood glucose levels, thereby forming a vicious, self-amplifying cycle that drives progressive metabolic deterioration. Accordingly, antioxidant-based therapeutics designed to specifically rebalance cellular redox status offer a pathophysiologically rational approach—capable of safeguarding β-cell viability and secretory capacity, reversing insulin resistance in metabolic tissues, and facilitating durable, physiology-aligned glycemic management.

In the diabetic rat model induced by streptozotocin (STZ), experimental doses were administered at 100–300 mg/kg, adjusted according to the specific protocol; the study included a blank control group, a disease model group, multiple treatment groups receiving graded doses of the test compound, and a positive control group treated with metformin, hyperglycemia suppresses the antioxidant defense system. This suppression results in a reduction in the levels of non-enzymatic antioxidants and a decline in the activities of antioxidant enzymes, thereby facilitating the accumulation of reactive oxygen species (ROS). The increased presence of ROS exacerbates oxidative stress, contributing to mitochondrial dysfunction and subsequent organ damage (Kaneko, 2016; Liu et al., 2019). Research has demonstrated that GLPs mitigate oxidative stress by scavenging hydroxyl radicals and superoxide anions, as well as by reducing the lipid peroxidation product malondialdehyde (MDA). This is achieved through the enhancement of antioxidant enzyme activities, including SOD, CAT, GPx, Mn-SOD, and GR. Additionally, GLPs lower plasma nitric oxide (NO) levels by inhibiting the mRNA expression of inducible nitric oxide synthase (iNOS), thereby alleviating oxidative damage and safeguarding pancreatic cells (Agarwal and Sohal, 1993; Huang et al., 2020). Furthermore, GLPs ameliorate insulin resistance by decreasing the homeostasis model assessment of insulin resistance index (HOMA-IR) (Xie et al., 2020). Electron microscopy observations have confirmed that GLPs can repair damage caused by oxidative stress, such as mitochondrial vacuolization and autophagosome formation, protect the ultrastructure of islet β-cell mitochondria, and normalize insulin secretion (Jia et al., 2008). Subsequent studies have demonstrated that GLPs can enhance inadequate insulin secretion in the advanced stages of type 2 diabetes. In both the oxidant intervention group (administered with ferrous sulfate) and the diabetic cohort, insulin levels initially increased before subsequently declining, whereas blood glucose levels persistently elevated (Senthilkumar et al., 2017). This suggests that oxidative stress exerts a dual influence on insulin secretion: moderate levels of oxidative stress may enhance insulin secretion, whereas excessive oxidative damage impairs the compensatory capacity of insulin secretion. Therefore, appropriate antioxidant treatment should be administered according to the different tolerance thresholds of various cells to oxidative stress (Kaneko, 2016; Li et al., 2019a; Liang et al., 2018; Pan et al., 2013a; Yang et al., 2009).

In summary, oxidative stress is a pivotal factor in the pathogenesis of diabetes, with excessive levels potentially leading to irreversible physiological damage. As an exogenous antioxidant, GLPs restore redox homeostasis by enhancing the levels of antioxidant enzymes and modulating the pancreatic microenvironment, thereby offering a novel theoretical foundation and potential therapeutic targets for treatment.

### 3.2 Inhibit the ectopic accumulation of lipids

Disruptions in lipid metabolism may result in the accumulation of lipid intermediates, including free fatty acids, diacylglycerol, and ceramides, in the liver, skeletal muscle, and adipose tissue. This accumulation can damage performance of pancreatic islet β-cells through lipotoxicity, exacerbate impaired insulin action, impair glucose metabolism, and ultimately lead to hyperglycemia and hyperinsulinemia, creating a vicious cycle. Dyslipidemia may exacerbate complications including cardiovascular and cerebrovascular diseases in individuals with diabetes, as well as contribute to the development of non-alcoholic fatty liver disease (NAFLD) and renal pathologies (Lv et al., 2019; Tong et al., 2019; Wu, 2018).

An environment characterized by elevated levels of glucose and free fatty acids (FFA) may contribute to the development of insulin resistance and promote apoptosis in pancreatic islet cells. The spontaneous type 2 diabetes animal model (db/db mice) was compared with a control group that did not receive GLPs drugs and a normal mouse group. GLPs have been shown to decrease concentrations of total cholesterol (TC), triglycerides (TG), low-density lipoprotein cholesterol (LDL-C), and FFA, while concurrently increasing high-density lipoprotein cholesterol (HDL-C) levels. Simultaneously, it suppresses the expression of critical lipolytic factors in adipose tissue, including hormone-sensitive lipase (HSL) and fatty acid-binding protein 4 (FABP4). It also downregulates the levels of sterol regulatory element-binding protein-1 (SREBP-1) and its downstream target, fatty acid synthase (FAS). Additionally, it activates the Janus kinase 2 (JAK2)/signal transducer and activator of transcription (STAT) signaling pathway and modulates the expression of stearoyl-CoA desaturase (SCD), which plays a role in lipid metabolism. These actions collectively contribute to the maintenance of blood lipid homeostasis and the reduction of hepatic lipid synthesis (Xiao et al., 2018). Subsequent research has demonstrated that the activation of the FAM3C-HSF1-CaM-AKT signaling pathway can ameliorate glucose and lipid metabolic disorders (Jia et al., 2023; Ren et al., 2021; Sun et al., 2015; Wang et al., 2023).

In-depth research shows that during the expansion of adipose tissue, hypoxia and apoptosis lead to macrophage recruitment, which exacerbates chronic inflammation and results in the secretion of various inflammatory cytokines. GLPs can improve inflammation-induced fat accumulation. Pro-inflammatory cytokines, particularly those belonging to the interleukin-1 (IL-1) family and tumor necrosis factor-alpha (TNF-α), can promote lipolysis and lipid transport by upregulating hormone-sensitive lipase (HSL) and inhibiting peroxisome proliferator-activated receptor gamma (PPAR-γ). This process exacerbates lipotoxicity and disrupts insulin signaling in peripheral tissues. GLPs diminish mRNA levels of inflammatory mediators, including IL-1β, TNF-α, and IL-6, within adipose tissue, reduce macrophage infiltration, significantly slow down chronic inflammation, thereby improving inflammation-induced lipolysis in adipose tissue, diminish its disruptive impact on the phosphorylation of insulin receptor substrate (IRS), and facilitate the restoration of the insulin signaling pathway. Simultaneously, GLPs effectively modulate blood lipid levels through diverse mechanisms, thereby mitigating fatty liver disease and rectifying the associated dysbiosis of the gut microbiota (Agarwal and Sohal, 1993; Liang et al., 2018; Sun et al., 2015; Xu et al., 2017; Yang et al., 2009; Zhu et al., 2013).

In conclusion, GLPs regulate lipid metabolism, promote insulin secretion, and improve insulin resistance. They can ameliorate associated with lipid metabolism disorders, and are expected to become new therapeutic targets for metabolic diseases.

### 3.3 Regulate glucose metabolism

**FIGURE 2:**
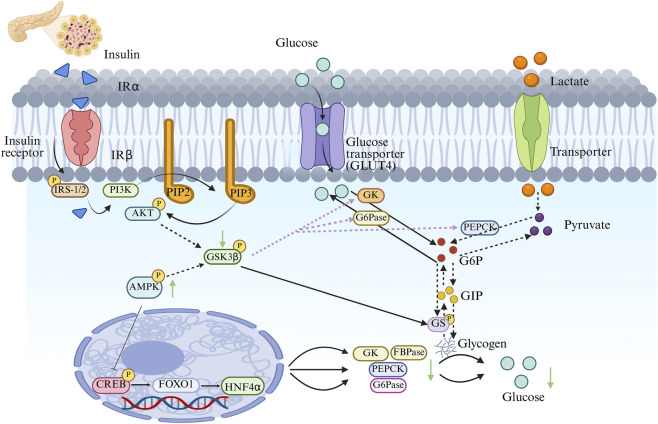
GLP regulates glucose metabolism homeostasis and improves the pathological process of diabetes.

Glucose metabolism is the core target for diabetes treatment. Its metabolic disorders can lead to uncontrolled blood glucose levels, accelerating the deterioration of β-cell function and leading to complications (Zhang and Lin, 2004) (Figure 2).

The spontaneous type 2 diabetes animal model (db/db mice) was used. The experimental groups included the group without GLPs drug administration, the normal mice group, and the metformin positive control group (administered 250 mg/kg) as the control. Research indicates that GLPs lower blood glucose levels by modulating the expression of genes encoding key enzymes involved in glucose metabolism. Lower blood sugar levels are connected to decreased activity of the rate-limiting enzyme in glycogenolysis, known as hepatic glycogen phosphorylase (GP), as well as a decrease in the mRNA expression levels of key enzyme genes related to gluconeogenesis, including glucose-6-phosphatase (G6Pase), fructose-1,6-bisphosphatase (FBPase), pyruvate carboxylase (PC), and phosphoenolpyruvate carboxykinase (PEPCK). GLPs suppresses glycogen synthase activity, elevates glucose-6-phosphate dehydrogenase (G-6-PD) levels, and decreases glucose production in the liver. At the same time, it upregulates the levels of pyruvate kinase (PK), phosphofructokinase-1 (PFK-1), and hepatic glucokinase (GK)—enzymes involved in glycolysis—while inhibiting glucose transporter 2 (GLUT2), promoting the translocation of GLUT4, enhancing glucose uptake by peripheral tissues, accelerating glucose metabolism, and preventing hyperglycemia. Further research has found that activating the JAK2 protein and the signaling route of AMPK can regulate gluconeogenesis and the breakdown and synthesis of fats, improve insulin signal transduction, and enhance insulin sensitivity. In-depth research reveals that the activation of the phosphatidylinositol 3-kinase (PI3K)/protein kinase B (Akt) signaling cascade increases the phosphorylation of glycogen synthase kinase 3β (GSK3β), boosts the production of glycogen in insulin-stimulated HepG2 cells, reduces blood sugar concentration levels, and improves insulin resistance (Han et al., 2016; Hikino et al., 1989; Liu and Ji, 2024; Ma et al., 2025; Pan et al., 2013a; Sun et al., 2015; Wu T. et al., 2022; Xiao et al., 2012; Yang et al., 2017).

In summary, GLPs regulate systemic glucose homeostasis via a multi-tiered, integrative mechanism: they inhibit excessive hepatic glucose production by downregulating key gluconeogenic enzymes and suppressing glycogenolysis; promote glucose catabolism in skeletal muscle and adipose tissue through AMPK- and JAK2-dependent glycolytic activation; and potentiate insulin action by enhancing phosphorylation cascades—particularly PI3K/Akt-mediated translocation of GLUT4 to the plasma membrane—ultimately increasing peripheral glucose disposal and metabolic efficiency. GLPs can not only directly improve glucose metabolism indicators but also repair the functions of metabolic organs, providing a fundamental treatment strategy for diabetes and becoming a key focus of future research.

### 3.4 Inhibit cell apoptosis

Pancreatic islet β cells undergo apoptosis due to hyperglycemia, lipotoxicity, oxidative stress, and inflammation, which in turn leads to insulin resistance and diminished insulin secretion. Meanwhile, they increase the apoptosis of cells in the kidneys, heart, vascular endothelium, nerves, and more, facilitating the emergence and progression of diabetes and its associated complications (Li et al., 2019b; Li et al., 2016; Zhang et al., 2017).

The investigation showed that Bcl-2 localizes to the outer mitochondrial membrane and inhibits apoptosis. (Ahagh et al., 2019). As a pro-apoptotic factor, the relative level of Bax to Bcl-2 determines the fate of cells (Ren B. et al., 2020). The research experiment utilized the STZ-induced diabetes mouse model. The designed groups were normal mice control, diabetes model control, and metformin positive control. GLPs modulate the Bax/Bcl-2 ratio by upregulating Bcl-2 and downregulating Bax protein expression, while also inhibiting pro-apoptotic genes including p53, caspase-3, and caspase-9 (Yang et al., 2019); and inhibit the apoptosis of pancreatic islet cells by triggering the PI3K/Akt pathway and regulating the MAPK/NF-κB apoptotic pathway, prevent mitochondrial dysfunction, repair pancreatic cell damage, and promote insulin secretion (Yang et al., 2009). Meanwhile, it markedly enhance manifestation of PDX-1, a essential gene that regulates pancreatic development and β-cell function, promotes the regeneration of pancreatic cells, and helps restore glucose homeostasis (Jiang et al., 2017; Pan et al., 2022; Shi et al., 2021; Yang et al., 2019; Yu et al., 2023).

Therefore, GLPs restore cell viability and alleviate pancreatic damage through anti-apoptosis combined with other mechanisms such as anti-oxidation and management of glucose and lipid metabolic processes, reverse hyperglycemia and its associated organ damage, fundamentally delay the progression of diabetes, and exert a three-dimensional protective impact on preventing and treating diabetes and its complications (Agarwal and Sohal, 1993; Li et al., 2019b; Liang et al., 2018; Yang et al., 2009; Zhu et al., 2013).

### 3.5 Repair pancreatic islet β cells

Repairing islet cells and promoting insulin secretion can restore the glucose-responsive secretory function of β-cells, immediately decrease blood sugar concentrations fasting and after eating; enhance insulin responsiveness in peripheral tissues like the liver, muscles, and fat tissue; inhibit hepatic gluconeogenesis and promote glucose utilization; and block glucotoxicity at its source, thereby establishing a positive feedback loop that stabilizes blood glucose levels and delays the progression of diabetes (Li et al., 2022; Li et al., 2009; Liang et al., 2020; Wang et al., 2016).

Mechanistically, GLPs protect islet cells from damage, reverse the decline in islet viability in diabetic mice, and increase serum insulin levels by reducing blood glucose, down-regulating blood lipids, inhibiting the production of unbound radicals, and reducing oxidation of lipids in the pancreas; elevating the levels of PDX-1 and Bcl-2 gene expression to prevent cell apoptosis; and improving disorders related to glucose and lipid metabolism and inflammatory responses by inhibiting relevant signaling pathways including NF-κB, PPARγ, and AMPK (Zhang et al., 2003). Further research has found that protein tyrosine phosphatase (PTP1B) is now being targeted as a new approach to fight type 2 diabetes and obesity. By dephosphorylating IRS, PTP1B leads to insulin resistance and irregularly affects the insulin signaling pathway. After GLPs intervention, the overexpression of PTP1B in liver tissue is inhibited, the phosphorylation of IRS at tyrosine residues is significantly improved, and insulin resistance is ameliorated (Pan et al., 2022; Yang et al., 2017; Yu et al., 2020; Yu et al., 2023; Zhang H. et al., 2024). Histological studies have confirmed that GLPs can partially protect β-cells from necrosis (β-cells almost disappear in the alloxan-treated group).

Therefore, GLPs act synergistically through multiple targets—such as antioxidative stress, anti-apoptosis, and improvement of glucose and lipid metabolic disorders—to protect pancreatic islet cells from damage, providing a new strategy for diabetes treatment (Zhu et al., 2013).

### 3.6 Suppress the inflammatory response and macrophage infiltration

**FIGURE 3:**
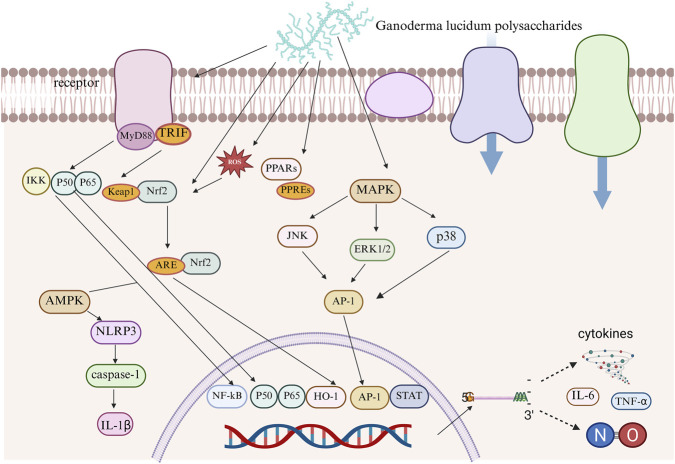
GLP improves diabetes through a multi-target anti-inflammatory pathway.

The inflammatory response is central to the onset and progression of diabetes. Persistent mild inflammation directly leads to disorders of blood glucose regulation and exacerbates complications such as diabetic nephropathy, cardiovascular diseases, and retinopathy through mechanisms such as impairing the function of pancreatic islet β cells, aggravating reduced sensitivity to insulin, and promoting vascular endothelial damage (Figure 3).

Research has found that apoptosis, oxidative stress, and disordered glycolipid metabolism can lead to increased macrophage recruitment and the development of chronic inflammatory responses, thereby stimulating the release of multiple inflammatory cytokines. By lowering the mRNA expression of inflammatory cytokines such as TNF-α, IFN-γ, IL-1β, and IL-6, reducing macrophage infiltration, and preventing inflammatory signal transduction, GLPs effectively mitigate chronic inflammation. Further investigations demonstrate that GLPs can prevent inflammation by interfering with the Toll-like receptor 4 (TLR4)/NF-κB signaling pathway. Therefore, the anti-inflammatory effect can act synergistically through multiple mechanisms to handle diabetes and the complications that arise from it (Gao et al., 2020; Li et al., 2019c; Wu et al., 2025; Xu et al., 2017; Zhu et al., 2013).

## 4 Mechanisms through which Ganoderma lucidum polysaccharides contribute to the prevention and treatment of diabetes

**FIGURE 4:**
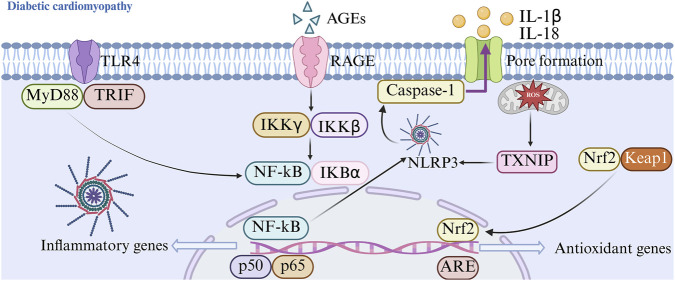
Mechanism of action of diabetes-related complications.

Long-term hyperglycemia in diabetic patients can cause numerous complications, significantly impacting their quality of life and safety (Shaw et al., 2009). This section systematically summarizes that GLPs can improve diabetic complications including diabetic kidney disease, liver injury from diabetes, heart disease due to diabetes, non-healing diabetic wounds, diabetic nerve disorders, diabetic eye conditions, and erectile dysfunction resulting from diabetes, through different pathways (Bi et al., 2024; Cole and Florez, 2020; Graves and Donaghue, 2019; Li et al., 2019c; Zhao et al., 2024) (Table 2; Figure 4).

**TABLE 2:** The principal actions of GLPs on diabetic liver injury, diabetic neuropathy, and the healing of refractory diabetic wounds.

Complications/Mechanisms	Diabetic liver injury (Downregulate AST, ALT)	Diabetic neuropathy	Refractory wounds in diabetes
Antioxidant	Upregulate SOD, GPX, CAT, Nrf2/Keap, Nrf2/HO-1 Downregulate ROS, MDA	Upregulate HO-1, NQO1, SOD, Nrf2 Downregulate ROS, MDA	Upregulate MnSOD, GPx-1 Downregulate p66 Shc, ROS, MDA
Anti-inflammatory	UpregulateSCFA Downregulate TNF-α, TLR4/NFκB	Upregulate IL-10 Downregulate IL-2,6, TNF-α,IFN-γ,NLRP3/NF-κB,MCP-1/C1q	Upregulate IL-10, M2 Downregulate M1, TNF-α, AGE
Anti-apoptosis	Upregulate Bcl-2 Downregulate Bax, Bax/Bcl-2	Upregulate Bcl-2 Downregulate caspase-3, Bax	Upregulate Bcl-2 Downregulate Bax, Bax/Bcl-2
Regulate carbohydrate and lipid metabolism	Upregulate PI3K/Akt, PPARγ/GLUT-4 Downregulate TC, TG, FBG	Downregulate TC, TG, FBG	Downregulate TC, TG, FBG
Repairing nerves	—	Upregulate FGFR1, ERK/AKT, NPC	—
Vasodilation	—	—	Upregulate iNOS, eNOS, NO

### 4.1 Diabetic nephropathy

Diabetic nephropathy (DN) is a significant complication associated with diabetes. Around 20%–30% of individuals with diabetes will experience diabetic nephropathy, and 20%–40% of these cases will advance to end-stage renal disease, a severe condition (Guan et al., 2021). The early clinical manifestation is microalbuminuria, indicating damage to the glomerular filtration barrier or dysfunction of tubular reabsorption. As the disease progresses, massive proteinuria occurs, causing an irreversible deterioration in kidney function and ultimately progressing to advanced kidney failure (Schernthaner and Schernthaner, 2013). Its pathogenesis is related to genetics, oxidative stress, lipid metabolism disorders, hemodynamic abnormalities, inflammatory responses, cell apoptosis, and renal fibrosis (Flemming et al., 2018). Currently, clinical treatment mainly aims to delay the progression of renal damage by regulating blood glucose, lipid, and blood pressure levels, yet it cannot halt or reverse the disease’s advancement. Consequently, discovering effective therapeutic drugs is essential.

The pathogenic nexus between diabetes and diabetic kidney disease (DKD) is fundamentally rooted in persistent hyperglycemia, which concurrently fuels three interconnected deleterious pathways: oxidative stress, low-grade inflammation, and accumulation of advanced glycation end products (AGEs). Collectively, these interconnected mechanisms cooperatively compromise the glomerular filtration barrier’s permselectivity and podocyte architecture, leading to escalating albuminuria, progressive tubulointerstitial scarring, and the eventual onset and advancement of diabetic nephropathy. A detailed mechanistic dissection of this pathogenic cascade not only elucidates the stepwise progression of diabetic nephropathy but also establishes a biologically informed framework for assessing polypharmacological strategies—such as GLPs.

The research experiments employed the STZ-induced diabetic mouse model or the db/db mouse model. The dosage of GLPs drugs could range from 100 to 300 mg/kg/day. The designed groups included the normal mice control group, the diabetic model control group, and the metformin positive control group. GLPs demonstrate notable protective properties for kidney function, effectively mitigating injury caused by conditions such as diabetic nephropathy and renal damage induced by toxic agents. Evidence indicates that GLPs enhance kidney function via several interrelated pathways: they reduce serum creatinine, blood urea nitrogen, and urinary albumin excretion in a dose-responsive manner; concurrently, they help regulate glycemic control and triglyceride metabolism, thereby slowing the advancement of renal complications. Mechanistically, GLPs exert renoprotective effects by suppressing the PI3K/Akt/mTOR signaling cascade, promoting autophagic—evidenced by increased Beclin-1 expression and LC3-II/LC3-I ratio, along with decreased p62 accumulation—while simultaneously attenuating apoptotic activity (reducing caspase-3 and caspase-9 levels) and dampening pro-inflammatory responses (lowering IL-6, IL-1β, and TNF-α). Moreover, they mitigate key structural abnormalities, including glomerular basement membrane thickening, mesangial matrix accumulation, and renal fibrosis. In addition, GLPs suppress both the upregulated expression of the (pro)renin receptor (PRR) and its cleaved soluble variant (sPRR), leading to diminished urinary renin activity and reduced angiotensin II (Ang II) concentrations—ultimately preventing pathological overactivation of the renin-angiotensin system (RAS). Moreover, GLPs suppress NOX4 expression, decrease H_2_O_2_ and MDA accumulation, and boost the enzymatic activity of key antioxidants—including SOD, CAT, and GSH-Px—thereby mitigating oxidative stress and associated DNA damage. Concurrently, they inhibit pro-inflammatory signaling axes such as COX-2, iNOS, and the TLR4/MyD88/NF-κB cascade, resulting in reduced inflammatory cell infiltration and attenuation of renal fibrosis. Polysaccharide fractions Ganoderma atrum polysaccharide (PSG-1) and Ganoderma applanatum polysaccharides (GAP)—derived from *Ganoderma lucidum* (tree-leaf reishi)—have been demonstrated to ameliorate acrylamide- or cadmium-induced nephrotoxicity, primarily through suppression of the oxidative DNA damage marker 8-hydroxy-2′-deoxyguanosine (8-OHdG), restoration of endogenous antioxidant enzyme function, and correction of dysregulated metabolic pathways (Fang et al., 2023a; Fang et al., 2023b; He et al., 2006; Hu et al., 2022; Jiang et al., 2021).

Further investigations have revealed that increased glucose levels can cause advanced glycation end-products (AGEs) to bind to their receptors (RAGE), which in turn activates NOX to produce reactive oxygen species (ROS). The generated ROS can trigger the Mitogen-activated protein kinase signaling route (MAPK), thereby activating p38 MAPK, ERK, and JNK, leading to glomerulosclerosis, renal interstitial fibrosis, damage caused by oxidative stress, pancreatic β-cell apoptosis, inflammatory responses, and increased collagen synthesis in cells. There is an interaction between the MAPK and NF-κB pathways that promotes the occurrence of inflammation. Meanwhile, the activation of MAPK may further upregulate the expression of NOX, forming a vicious cycle and aggravating the development of nephropathy. Experimental results in a controlled environment reveal that GLPs can markedly suppress the proliferation of HBZY-1 cells, reduce the expression of NOX1 and NOX4 as well as pro-fibrotic proteins, prevent buildup of type IV collagen and AGEs, significantly alleviate injury due to kidney fibrosis, and enhance renal function (Pan et al., 2013b; Pan et al., 2023). In-depth research has revealed that the TGF-β1/Smad signaling route is widely acknowledged for its significant role in the development of renal fibrosis in DKD. By inhibiting the TGF-β1/Smad pathway, GLPs help maintain the balance between the synthesis and degradation of the extracellular matrix (ECM), thereby suppressing glomerular mesangial cell proliferation and renal fibrosis. By inhibiting the PI3K/Akt/mTOR signaling pathway, GLPs also reduce inflammation, oxidative stress, apoptosis, and autophagy inhibition. Through these aforementioned mechanisms, GLPs enhance kidney function markers like serum creatinine and blood urea nitrogen, safeguarding the kidneys from harm (He et al., 2006; Hu et al., 2022; Yang et al., 2019).

In conclusion, GLPs maintain the homeostasis of the renal microenvironment and alleviate renal fibrosis through multiple targets, such as anti-oxidation, anti-inflammation, anti-apoptosis, regulation of lipid metabolism, and blocking relevant pathways, formulating a theoretical framework to support the research and creation of novel pharmacological treatments for nephropathy.

### 4.2 Diabetic liver injury

GLPs demonstrate robust hepatoprotective activity across a spectrum of liver pathologies—ranging from acute hepatotoxicity and NAFLD to progressive fibrosis and chemically induced hepatic injury. Their therapeutic efficacy stems from pleiotropic molecular actions, including suppression of oxidative stress, attenuation of pro-inflammatory cytokine cascades, inhibition of mitochondrial- and death receptor–mediated apoptosis, and restoration of lipid and glucose homeostasis through modulation of key metabolic regulators (Radziuk and Pye, 2001).

In chemically induced liver injury models—including carbon tetrachloride (CCl_4_), acetaminophen (APAP), cadmium (Cd), and alcohol (AA) exposure—GLPs activate the Nrf2–Keap1 antioxidant axis, leading to nuclear translocation of Nrf2 and subsequent upregulation of heme oxygenase-1 (HO-1) and downstream phase II enzymes. This results in enhanced activities of SOD, CAT, and GSH-Px, reduced levels of ROS, MDA, and the oxidative DNA lesion marker 8-hydroxy-2′-deoxyguanosine (8-OHdG) (Li et al., 2020). Concurrently, GLPs suppress innate immune hyperactivation by inhibiting both the NLRP3 inflammasome assembly and the TLR4–MyD88–NF-κB signaling cascade, thereby significantly downregulating key pro-inflammatory mediators such as IL-1β, IL-6, and TNF-α (Liu J. et al., 2025; Zhang Y. et al., 2025). Moreover, GLPs exert hepatoprotective effects via the gut–liver axis by modulating dysbiotic gut microbiota composition, reinforcing intestinal epithelial integrity—including tight junction protein expression, and reducing bacterial translocation and endotoxin leakage—thereby attenuating hepatic inflammation and injury (Zhang N. et al., 2024; Zhang X.-T. et al., 2024; Zhang Y. et al., 2025; Zhu et al., 2016).

In experimental liver fibrosis models, GLPs impede disease progression by concurrently suppressing two pivotal profibrogenic signaling axes: the TLR4–MyD88–NF-κB cascade—central to inflammation-driven fibrogenesis—and the canonical TGF-β–Smad pathway, a master regulator of extracellular matrix production. This dual inhibition effectively blocks the transdifferentiation and activation of quiescent hepatic stellate cells (HSCs) into collagen-secreting myofibroblasts, markedly decreasing deposition of fibrillar collagen type I (Col I) and α-smooth muscle actin (α-SMA), while also triggering cell cycle arrest in activated HSCs—thereby halting fibrotic expansion. The newly developed GLP–MnO_2_ nanozyme integrates intrinsic catalase (CAT) and superoxide dismutase (SOD)-mimetic activities with selective hepatic accumulation and real-time MRI contrast capability, thereby enabling synergistic antioxidant intervention and non-invasive monitoring—significantly amplifying its antifibrotic efficacy *in vivo* (Chen et al., 2008; Jiang et al., 2021; Li et al., 2007).

In metabolic liver disorder models—including high-fat diet (HFD)-induced obesity, NAFLD, and T2DM—GLPs ameliorate hepatic oxidative stress via the Nrf2/HO-1 pathway. They concurrently suppress NF-κB–driven transcription of pro-inflammatory genes such as *Tnfa* and *Il1b*, and modulate bile acid and lipid homeostasis through activation of the farnesoid X receptor (FXR)–small heterodimer partner (SHP) axis. This leads to transcriptional repression of lipogenic regulators—including sterol regulatory element-binding protein 1c (SREBP1c), fatty acid synthase (FAS), and acetyl-CoA carboxylase (ACC)—resulting in reduced hepatic steatosis, improved insulin sensitivity, and systemic metabolic restoration. In hyperlipidemia-associated hepatic steatosis, GLPs exert potent hypolipidemic effects—markedly lowering serum TC, TG, and LDL-C, while elevating HDL-C—thereby mitigating lipid accumulation and structural damage in hepatocytes. In radiation-induced liver injury, GLPs preserve mitochondrial redox balance in a dose-dependent manner by sustaining the activity of key antioxidant enzymes, outperforming the classic antioxidant α-tocopherol in both efficacy and mitochondrial specificity (Cefalu and Hu, 2004). GLPs reduce the activities of serum aspartate aminotransferase (AST) and alanine aminotransferase (ALT), which indicate liver damage, and elevate the serum total protein (TP) and albumin (ALB) levels, which reflect liver’s synthetic function, thereby helping to repair liver injury (Chang et al., 2013; Chen C. et al., 2022; Chen S. et al., 2022; Dong et al., 2024; Fan et al., 2025; Fang et al., 2025; Xiao et al., 2017; Zheng et al., 2021).

In summary, GLPs work via multiple pathways, including enhancing disorders of glucose and lipid metabolism, antioxidation, anti-inflammation, anti-apoptosis, improving insulin resistance, and repairing the intestinal barrier. It targets and regulates multiple signaling pathways through the gut-liver axis. GLPs have broad clinical application prospects in the treatment of diabetes-related liver diseases, NAFLD, liver fibrosis, and acute liver injury (Zhang Y. et al., 2025).

### 4.3 Diabetic cardiomyopathy

**FIGURE 5:**
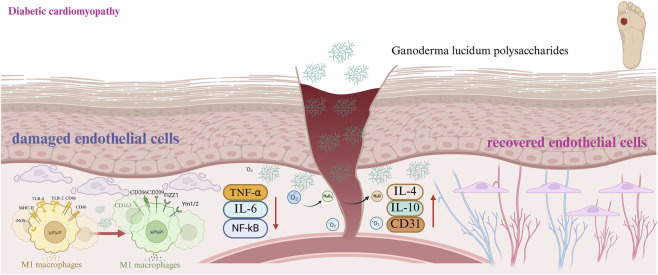
Pathological causes of diabetic cardiomyopathy.

Extensive evidence supports the broad-spectrum cardioprotective properties of GLPs, which effectively counteract diverse cardiac pathologies—such as pathological myocardial hypertrophy, viral or autoimmune myocarditis, atherosclerotic plaque development, sepsis-associated myocardial depression, ischemia-reperfusion injury, and cardiotoxicity triggered by chemotherapeutic agents (Ubaidillah et al., 2016; Wang Y. et al., 2025) (Figure 5).

In experimental models of pathological myocardial hypertrophy, GLPs exert protective effects by upregulating the PPARγ/PGC-1α signaling axis. This activation attenuates angiotensin II (Ang II)–driven cardiac remodeling—evidenced by downregulated expression of hypertrophic markers (ANP, BNP, and β-MHC), improved systolic function in transverse aortic constriction (TAC) mice (increased ejection fraction [EF%] and fractional shortening [FS%], alongside reduced left ventricular internal diameter [LVID] and heart weight-to-body weight ratio [HW/BW]), and suppression of fibrotic progression (decreased expression of Col1a1, Col3a1, fibronectin, and α-smooth muscle actin [α-SMA]). In murine models of myocarditis, Ganoderma atrum polysaccharide (PSG) exerts cardioprotection by directly interacting with TLR4, thereby suppressing TLR4-driven oxidative stress and inflammation—specifically through inhibition of the NF-κB/NLRP3 inflammasome pathway and disruption of the IRF1/VEGFA/14-3-3γ signaling axis. Concurrently, PSG preserves mitochondrial integrity by stabilizing membrane potential and interrupting the ROS/NLRP3–mediated apoptotic cascade. Notably, this protective effect mitigates PD-1 inhibitor–associated cardiotoxicity without compromising antitumor immunity. Regarding atherosclerosis, psoralen-associated polysaccharide–peptide conjugates suppress foam cell formation and attenuate perivascular adipose tissue accumulation (Zhen et al., 2023).

In sepsis-induced cardiac dysfunction, GLPs activate the SIRT1 pathway—a effect that is abolished by the selective SIRT1 inhibitor EX-527—leading to marked anti-inflammatory actions (reduced levels of IL-1α, IL-6, TNF-α, CK-MB, LDH, and diminished inflammatory cell infiltration), inhibition of cardiomyocyte apoptosis (downregulation of caspase-3, caspase-9, and Bax; decreased TUNEL-positive nuclei), and stimulation of myocardial cell proliferation (elevated expression of PCNA and cyclin D1) (Xu et al., 2021). In models of myocardial ischemia-reperfusion injury, selenium-fortified *Ganoderma lucidum* polysaccharide (Se-GLP) significantly boosts the enzymatic activity of endogenous antioxidants—including SOD, CAT, and GSH-Px—and elevates overall antioxidant capacity, resulting in marked suppression of oxidative stress biomarkers such as MDA and intercellular adhesion molecule-1 (ICAM-1) (Ashriyah et al., 2015; Shi et al., 2010).

In doxorubicin-induced cardiotoxicity, PSG-1 confers cardioprotection by selectively suppressing the mitochondrial intrinsic apoptotic pathway—evidenced by enhanced MnSOD activity, preservation of mitochondrial membrane potential, inhibition of mitochondrial permeability transition pore (mPTP) opening, and attenuated cytochrome c release into the cytosol (Chen Y.-S. et al., 2019; Ubaidillah et al., 2016). Collectively, GLPs demonstrate robust therapeutic potential against a spectrum of cardiac disorders—including diabetic cardiomyopathy—by engaging multiple complementary mechanisms: activation of the PPARγ/PGC-1α axis to improve mitochondrial biogenesis and energy metabolism; suppression of the TLR4/NF-κB/NLRP3 inflammatory cascade; preservation of mitochondrial structural and functional integrity; upregulation of SIRT1-mediated deacetylation signaling; and potentiation of endogenous antioxidant defenses and anti-apoptotic pathways. These multifaceted actions underscore their promising translational utility in cardiovascular medicine (Mu et al., 2025; Wang J.-H. et al., 2025).

The research experiment employed high-fat diet and STZ-induced diabetic mouse models or db/db mouse models. The dosage of GLPs drugs could range from 100 to 400 mg/kg/day (set according to the size of the mice and in accordance with ethical standards). The designed groups included normal mice control, diabetic model control, and metformin positive control. Accumulating preclinical and clinical data strongly implicate gut microbial imbalance as a key contributor to the onset and progression of diabetic cardiomyopathy. Sustained hyperglycemia perturbs host–microbe metabolic crosstalk which in turn destabilizes microbial community structure and weakens tight junction protein expression, culminating in enhanced gut epithelial permeability. This compromised barrier facilitates the systemic translocation of immunostimulatory microbial derivatives—particularly endotoxin (LPS) and pro-fibrotic oxidized trimethylamine N-oxide (TMAO)—which drive cardiac fibroblast activation, collagen deposition, and adverse myocardial remodeling. In contrast, short-chain fatty acids (SCFAs) confer broad cardiovascular protection through anti-inflammatory, anti-fibrotic, and metabolic regulatory mechanisms. Importantly, GLPs exert gut-heart axis–mediated benefits by selectively enriching SCFA-producing bacterial taxa, boosting colonic SCFA bioavailability, and suppressing hepatic TMAO synthesis—thereby mitigating key drivers of diabetic cardiomyopathy progression.

Long-term diabetes can lead to an increase in myocardial collagen fibers and the degree of collagen cross-linking, which will aggravate myocardial fibrosis, make the myocardium stiffer, and raise the likelihood of heart conditions like heart attack and high blood pressure (Flier et al., 1988). GLPs improve myocardial fibrosis by reducing the levels of AGEs and oxidative stress induced by hyperglycemia and hyperlipidemia (Lorenzi, 1992). AGEs accumulate in myocardial tissue and create covalent bonds with substances like collagen, causing an increase in collagen formation, aggravated myocardial fibrosis, and promotion of aortic sclerosis. AGEs can also bind to their receptor (RAGE), activating downstream pro-fibrotic and pro-inflammatory signaling pathways. In addition, diabetic patients exhibit significant oxidative stress. ROS and AGEs interact to form a vicious cycle. ROS serves as a common upstream event in pathophysiological pathways such as AGE formation and protein kinase C (PKC) activation during diabetic complications. ROS promotes the formation of AGEs and upregulates the expression of RAGE by triggering the NF-κB pathway, thereby boosting biological effects of AGEs (Brownlee, 2005; Gopal and Indira, 2009; Guo et al., 2024). The interaction between AGEs and RAGE triggers NOX, which boosts ROS generation. As an AGEs breaker, GLPs are capable of minimizing the buildup of AGEs in the myocardium which obstructing AGEs-RAGE axis, thereby enhancing the solubility of collagen and reducing fibrotic structural damage. The functions of enzymes that act as antioxidants such as SOD, GSH-Px, and CAT in the myocardium are enhanced, Nrf2 expression is increased, and ROS and MDA production levels are lowered, thereby strengthening the endogenous antioxidant defense, blocking the AGEs-ROS cycle, and thus improving cardiac function (Meng et al., 2011; Song et al., 2021). Further studies have shown that GLPs have the ability to boost levels of PI3K, p-Akt, eNOS, and NO inside the aorta, thereby significantly improving endothelium-dependent aortic diastolic function and alleviating endothelial dysfunction (Zhu et al., 2014).

In summary, after long-term administration of GLPs, by maintaining glucose and lipid metabolism homeostasis, exerting antioxidative, inflammation-reducing, anti-fibrotic impacts, and regulating gastrointestinal-heart axis, the physiological state and pathological findings in rats remained normal, with no obvious organ damage observed, indicating good safety. These effects can synergistically improve the pathological damage associated with cardiomyopathy, presenting an innovative strategy to prevent and treat complications (Li W.-J. et al., 2018; Wicaksono et al., 2016).

### 4.4 Refractory diabetic wounds

**FIGURE 6:**
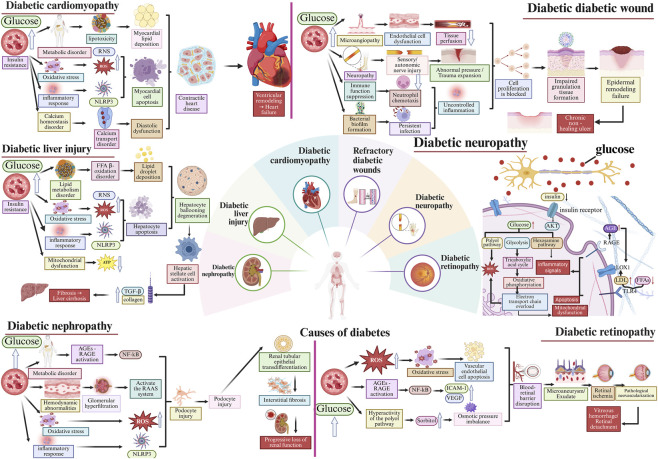
Pathological reasons for the difficult healing of diabetic wounds.

Diabetic refractory wounds are serious complications. Due to limited treatment options, they often lead to amputation. Factors such as inflammation, reduced granulation tissue formation, neuropathy, and insufficient angiogenesis impede wound healing, and oxidative stress is a key pathogenic factor (Brem and Tomic-Canic, 2007). Hyperglycemia causes excessive production of superoxide anions (O_2_^−^), activates pathways such as PKC, polyol, and hexosamine, and increases the formation of AGEs. Ultimately, it leads to increased vascular permeability, defective angiogenesis, and activation of pro-inflammatory pathways, which finally hinders wound healing (Fadini et al., 2010; Giacco and Brownlee, 2010) (Figure 6).

Studies have identified mitochondria as primary origin of ROS in diabetes (Huang et al., 2006). The research experiment employed a high-fat diet and STZ-induced diabetic wound model. The dosage of GLPs drugs was 50–300 mg/kg/day or was applied topically (based on the size of the mice and in accordance with ethical standards). The designed groups included normal mice as the control and the diabetic model as the control. GLPs exert antioxidant effects and promote angiogenesis through the following pathways: through increasing the performance of antioxidant enzymes including SOD, CAT, MnSOD, and GPx-1, by inhibiting tyrosine nitration of MnSOD to restore its enzyme activity; in addition, by suppressing the representation of pro-oxidant protein p66Shc as well as its phosphorylation at the Ser36 site, thereby diminishing the generation of ROS and MDA along with a rise in NO levels. GLPs repair pancreatic islet cell function by regulating abnormalities in glucose and lipid metabolism. By regulating eNOS expression and enhancing activity of iNOS, GLPs increase NO levels in the skin, thereby repairing damage to the microvascular endothelium and improving blood supply (Cao et al., 2014; Ceriello, 2006; Khosravifar et al., 2023; Redondo-Horcajo et al., 2010; Tie et al., 2009; Tie et al., 2012).

GLPs accelerate wound closure via a pleiotropic, multi-target mechanism—spanning the stimulation of keratinocyte and fibroblast proliferation and motility, modulation of key signaling cascades, suppression of excessive inflammatory responses, mitigation of oxidative damage, and enhancement of therapeutic efficacy via advanced delivery platforms such as nanocarriers and stimuli-responsive hydrogels. At the cellular level, GLPs exert concentration-dependent stimulatory effects on the proliferative and migratory capacities of both dermal fibroblasts and epidermal keratinocytes. Notably, treatment with GLPs at low concentrations (0.1–0.2 μg/mL) markedly improves viability in human monocytic THP-1 cells and robustly enhances directional migration of murine NIH/3T3 fibroblasts. At concentrations ranging from 10 to 40 μg/mL, GLPs activate the canonical Wnt signaling cascade, leading to enhanced nuclear translocation and stabilization of β-catenin. Concurrently, they stimulate the production of transforming growth factor-beta 1 (TGF-β1) and the C-terminal propeptide of type I collagen (CICP)—biomarkers indicative of active collagen synthesis—thereby facilitating extracellular matrix deposition, maturation, and dynamic tissue remodeling during wound healing. In rat intestinal epithelial IEC-6 cells, GLPs enhances proliferative capacity, directional motility, and functional maturation—processes mechanistically linked to the marked upregulation of ornithine decarboxylase (ODC) and the proto-oncogene c-Myc, key regulators of polyamine biosynthesis and cellular differentiation (Hu et al., 2018; Sun et al., 2011; Zhao et al., 2021).

Regarding anti-inflammatory activity, the oxidized GLP–carboxymethyl chitosan–based hydrogel (designated G-GLP) combines superior mechanical integrity with outstanding biocompatibility. It orchestrates immunomodulation by skewing macrophage differentiation toward the pro-resolving M2 phenotype, suppressing pro-inflammatory M1 activation, and scavenging intracellular reactive ROS. Collectively, these actions mitigate chronic inflammation, expedite revascularization, and enhance organized collagen synthesis and deposition. Regarding antioxidant functionality, GLPs demonstrate potent capacity to scavenge reactive oxygen species, as evidenced by high oxygen radical absorbance capacity (ORAC) values. Furthermore, selenium-enriched GLP (Se-GLP) effectively attenuates oxidative damage in ischemia–reperfusion injury by significantly lowering levels of MDA and intercellular adhesion molecule-1 (ICAM-1), while concurrently restoring intracellular glutathione (GSH) pools and reactivating key antioxidant enzymes—including SOD, CAT, and GSH-Px (Zhang et al., 2019; Zou et al., 2025).

For the management of complex, chronic wounds, multifunctional yolk–shell nanoparticles (YSPs) engineered to encapsulate GLP integrate synergistic antibacterial action, ROS-scavenging capability, and near-infrared (NIR)-responsive photothermal conversion. Critically, their unique architecture enables spatiotemporally controlled GLP release—making them highly suitable for combined wound healing therapies, including laser-triggered on-demand delivery and photothermal-enhanced tissue regeneration. *In vivo* experiments in murine full-thickness excisional wound models demonstrate that topical administration of GLPs at 10–40 mg/mL markedly accelerates wound closure kinetics and reduces overall healing duration. The mechanism is related to the activation of the Wnt/β-catenin pathway and the upregulation of TGF-β1.

In conclusion, GLPs hold substantial translational potential for managing diverse wound pathologies—including diabetic ulcers, thermal burns, and intestinal mucosal damage—by orchestrating a coordinated, multi-modal therapeutic response: stimulating epithelial and stromal cell proliferation/migration; fine-tuning regenerative signaling axes (e.g., Wnt/β-catenin and TGF-β1); reprogramming macrophage phenotypes toward pro-healing M2 states; bolstering endogenous antioxidant defenses; and leveraging engineered delivery platforms for spatiotemporally controlled bioactivity.

Further research has found that hydrogels have attracted considerable focus in the biomedical sector owing to superior biocompatibility, remarkable water absorption and retention capabilities (such as absorbing exudate and preventing infection), as well as plasticity and tissue adhesion (enabling them to conform to wounds and facilitate sustained drug release). A new type of double-network hydrogel, OGLP-CMC/SA, was designed using oxidized GLP as the structural base. This hydrogel enhances proliferation and migration of fibroblasts; improves antibacterial performance; boosts antioxidant capacity by increasing the performance of enzymes with antioxidant properties, breaking vicious cycle between ROS and AGEs; and alleviates inflammatory response by facilitating transition of M1 macrophages, which are pro-inflammatory, to M2 macrophages, which are anti-inflammatory, pro-inflammatory factors TNF-α and IL-6 showed decreased levels, whereas anti-inflammatory factor IL-10 showed an increase. Therefore, through properties such as regulating M2 polarization, antioxidant activity, antibacterial effects, as well as properties that combat inflammation, this hydrogel enhances the phagocytic ability of macrophages, promotes epidermal growth and collagen deposition, and offers a potential strategy for treating persistent diabetic wounds (Li et al., 2024). Recently, a novel thermosensitive hydrogel system has been engineered by integrating uncoated nanoparticles composed of Ganoderma lucidum polysaccharides and ferulic acid (GFNPs) with recombinant human epidermal growth factor (rhEGF). GFNP is fabricated via a precipitation-based synthesis approach and exhibit multifunctional bioactivities, including potent antioxidant capacity, induction of M2-like macrophage polarization, and broad-spectrum antibacterial effects. When co-delivered with rhEGF, they act synergistically to amplify fibroblast and endothelial cell proliferation, directional migration, and capillary-like tube formation—key processes underlying tissue vascularization and wound repair. In diabetic mice, this hydrogel effectively dampens inflammatory signaling, enhances blood vessel formation, and facilitates comprehensive tissue regeneration—positioning it as a compelling, integrated wound dressing approach for the clinical management of diabetic ulcers (Li et al., 2026).

In conclusion, GLPs promote the healing of diabetic wounds primarily through mechanisms including antioxidant, decreases inflammation, and antibacterial effects, along with by regulating disordered glucose and lipid metabolism and promoting angiogenesis, thereby providing a theoretical basis for research on diabetic wound healing.

### 4.5 Diabetic neuropathy

GLPs exhibit broad-spectrum neuroprotective properties across a range of neurological disorders—such as Alzheimer’s disease (AD), spinal cord injury (SCI), cerebral ischemia, epilepsy, major depressive disorder, and neuroinflammatory conditions. These beneficial effects arise through diverse, interconnected molecular and cellular pathways (Liu et al., 2022). The research experiment employed *in vitro* models of microglia and nerve injury (including zebrafish *in vivo* experiments, cerebral ischemia rat models, neuronal injury models, Alzheimer’s disease 5xFAD mouse models, and spinal cord injury rat models). The drug dosage was set according to the size of the mice and in accordance with ethical standards. The designed groups were the sham operation group/normal mouse control group, and the model control group.

In the Alzheimer’s disease model, GLPs activate FGFR1 and its downstream ERK/AKT pathways, promoting the proliferation of Ki67^+^SOX2^+^ hippocampal neural progenitor cells and the generation of BrdU^+^NeuN^+^ new neurons. At the same time, they reduce Aβ deposition in the brains of APP/PS1 mice, thereby improving the cognitive function of APP/PS1 mice. Regarding neuroinflammatory processes, GLPs exert potent anti-inflammatory effects by modulating microglial activation. Specifically, they suppress the production of key pro-inflammatory mediators—including IL-1β, IL-6, and iNOS—in a concentration-dependent manner, while concurrently upregulating anti-inflammatory markers such as transforming growth factor-beta (TGF-β) and arginase-1 (Arg1) (Seweryn et al., 2021). Moreover, GLPs impede microglial motility, prevent activation-associated morphological remodeling, and dampen phagocytic capacity (Cai et al., 2017). Importantly, they also downregulate Aβ-induced expression of complement component C1q, thereby mitigating C1q-dependent synaptic pruning and preserving synaptic integrity (Guo et al., 2017; Huang et al., 2017).

In experimental spinal cord injury models, GLPs preserve myelin ultrastructure and enhance functional recovery through multimodal neuroprotective actions: they suppress neuronal and oligodendroglial apoptosis—evidenced by reduced caspase-3 cleavage—attenuate neuroinflammation via downregulation of TNF-α and myeloperoxidase (MPO) activity, and counteract oxidative damage by decreasing MDA and NO levels while boosting SOD activity. Notably, the capacity of GLPs to safeguard myelin integrity surpasses that of methylprednisolone, a standard clinical corticosteroid used in acute SCI management. In preclinical models of cerebral ischemia, GLPs significantly diminish cerebral infarct volume and attenuate neuronal cell death by modulating the mitochondrial apoptotic pathway—specifically, by upregulating the anti-apoptotic protein Bcl-2, downregulating the pro-apoptotic factor Bax, and suppressing the activation of executioner caspases (caspase) (Guo et al., 2017; Liu X. et al., 2025; Zhou et al., 2010). In rodent models of epilepsy, GLPs confer neuroprotection primarily by preventing pathological intracellular calcium accumulation and normalizing the expression levels of calcium/calmodulin-dependent protein kinase II alpha (CaMKIIα) (Jiang et al., 2024; Wang et al., 2018). Furthermore, the GLPs analog GCPS-2 has been shown to mitigate kainic acid (KA)-induced seizure activity via dual mechanisms—suppressing neuronal apoptosis and dampening neuroinflammatory responses (Chen Y. et al., 2025; Gokce et al., 2015; Li H. et al., 2021; Wang et al., 2014; Zhou et al., 2010).

In preclinical models of depression, GLPs produce rapid-onset and sustained antidepressant effects via a Dectin-1 receptor–mediated pathway. This involves suppression of hippocampal microglial activation and astrocytic hyperplasia, restoration of cytokine homeostasis—shifting from a pro- to an anti-inflammatory milieu—and enhancement of brain-derived neurotrophic factor (BDNF) expression and AMPA receptor functionality. Moreover, GLPs influence brain health indirectly through gut–brain axis modulation: they reshape the intestinal microbiota composition—enriching *Lactobacillus* species and lowering the Firmicutes-to-Bacteroidetes (F/B) ratio—and elevate circulating and colonic levels of neuroactive SCFAs (Chong et al., 2025; Huang et al., 2017; Zhang et al., 2018). These microbial and metabolic shifts collectively suppress NLRP3 inflammasome activation and downstream NF-κB signaling in the CNS, resulting in attenuated neuroinflammation and enhanced cognitive performance.

This section clarifies that GLPs repair nerve damage and alleviate cognitive impairment in mice through mechanisms such as regulating the host immune response, antioxidation, anti-apoptosis, and promoting nerve regeneration. It is beneficial to introduce a fresh pathway for clinical implementation in managing pertinent nerve injuries.

### 4.6 Diabetic retinopathy

Extensive evidence demonstrates that GLPs confer broad-spectrum neuroprotective and anti-ischemic effects in retinal ischemia–reperfusion (RIR) injury—a key pathophysiological mechanism underlying both diabetic retinopathy and glaucoma (Zhu et al., 2025).

Research indicates that GLP administration exerts multifaceted retinal protection in RIR injury: structurally, it preserves retinal architecture by mitigating thinning—particularly in the inner plexiform and ganglion cell layers—and significantly suppressing apoptosis, as evidenced by reduced TUNEL-positive cell counts; mechanistically, it upregulates the Nrf2/HO-1 antioxidant axis, thereby boosting superoxide dismutase (SOD) activity and lowering malondialdehyde (MDA) accumulation; functionally, it restores electrophysiological integrity, reflected in enhanced amplitudes across all major ERG waveforms (a-wave, b-wave, and oscillatory potentials), suggesting preserved functionality of photoreceptors, bipolar cells, and retinal ganglion cells. Moreover, GLPs attenuate pathological reactive gliosis—evidenced by suppressed activation of GFAP-expressing astrocytes and IBA-1-positive microglia—and help reestablish glia–neuron crosstalk through the modulation of key intercellular communication proteins, including connexin 43 (Cx43) and aquaporin-4 (AQP4). Regarding vascular integrity, GLPs exert protective effects by: (i) inhibiting aberrant neovascularization through downregulation of the HIF-1α/VEGF/Notch signaling axis; (ii) enhancing endothelial barrier function via upregulation of vascular endothelial cadherin (VE-cadherin) and endothelial nitric oxide synthase (eNOS), thereby reinforcing intercellular adherens junctions; and (iii) promoting microvascular perfusion by increasing functional capillary density. Regarding immunomodulation, GLPs drive microglial phenotypic switching from the pro-inflammatory M1 state toward the tissue-reparative M2 phenotype—evidenced by decreased secretion of IL-1β and TNF-α, alongside elevated levels of IL-4 and IL-10. This anti-inflammatory reprogramming is mechanistically linked to suppression of the JAK2/STAT3 signaling cascade (Long et al., 2025).

Collectively, GLPs show strong therapeutic efficacy against retinal pathologies induced by ischemia–reperfusion injury, acting via a multi-faceted pharmacological profile that includes antioxidant, anti-inflammatory, anti-apoptotic, anti-angiogenic, and gliosis-modulating activities.

### 4.7 Erectile dysfunction induced by diabetes

The latest research has found that erectile dysfunction is a prevalent complication among individuals with diabetes, but the existing treatment options are limited. The research experiment utilized a diabetic ED rat model, and the designed groups were the normal control group and the model control group. GLPs protect the structural integrity of the cavernous endothelium by mitigating oxidative pressure within corpus cavernosum; they minimize programmed cell death by hindering phosphorylation of the ERK/JNK pathway, blocking transcription and translation of apoptosis-related proteins like caspase-3 and Bax, while also elevating levels of Bcl-2 factor, and maintaining mitochondrial membrane potential stability; they promote vasodilation by enhancing NOS activity, promoting NO production, increasing cyclic guanosine monophosphate (cGMP) levels, maintaining NO-cGMP pathway activity, down-regulating arginase II protein expression, and reducing competitive consumption of L-arginine; they promote tissue repair and fibrosis remodeling by up-regulating TGF-β1. This was the first study to demonstrate that GLPs improve diabetes mellitus-induced erectile dysfunction (DMED) through three mechanisms: anti-oxidation, anti-apoptosis, and vascular repair This finding offers fresh proof for utilizing GLPs in treating diabetic complications (Yao et al., 2022).

## 5 Ganoderma lucidum polysaccharides regulate diabetes via the gut microbiota

The gut microbiota can exert either beneficial or detrimental effects on host health. An increasing amount of research points to the critical role of microbiota imbalance in the onset and development of diabetes. Research has found that GLPs regulate the microbial communities inside the mouth, intestines, pancreas, and lungs of rats suffering from T2DM by significantly increasing abundance of beneficial bacteria such as *Lactobacillus*, *Bifidobacterium*, *Prevotella*, *Blautia*, selenium-rich bacteria, along with *Coprococcus*, in addition to by inhibiting harmful bacteria such as *Staphylococcus*, *Escherichia*, *Ruminococcus torques (Holdeman and Moore 1974)*, *Streptococcus mutans (Clarke 1924)*, and *Clostridium perfringens (Veillon and Zuber 1898)*, consequently bettering glucose metabolism disturbances and alleviating reduced sensitivity to insulin (Li M. et al., 2021; Ruiz et al., 2023; Sang et al., 2021; Yu et al., 2024).

However, the gut microbiota’s composition determines how intestinal barrier functionality and directly impacts progression of diminished response to insulin and obesity. GLPs raise positive microbes’ levels including *Lactobacillus* and *Bifidobacterium*, reducing the release of lipopolysaccharides and endotoxins into the bloodstream caused by pro-inflammatory bacteria like *Actinobacteria*. It also increase probiotics’ plentiful presence like *Prevotella* and *Parasutterella*, enhance relative plenty of *Enterococcus* species associated with anti-diabetic effects, along with stimulate proliferation of bacteria that produce butyrate like *Clostridium butyricum (Prazmowski 1880)*. Furthermore, GLPs activate GPR43 pathway and promote creation of short-chain fatty acids (SCFAs). By regulating bacteroidetes to Firmicutes ratio (B/F), a crucial metric for assessing gut microecological equilibrium, GLPs restore it to a level close to normal (changes in the B/F value are closely related to various sickness like diabetes, intestinal inflammation disorder, together with breast malignancy). Therefore, through the above-mentioned ways of regulating the microbiota-metabolism axis, GLPs restore carbohydrate metabolism, augment insulin responsiveness, reduce inflammation, reduction in blood sugar levels, and restore gut barrier function (Chen M. et al., 2019; Shao et al., 2021; Xu et al., 2017; Yahya et al., 2019).

In previous studies, it has been found that there is a series of interactions among the microbiome within mouth, intestines, pancreas, together with lungs, which makes the occurrence and development of intestinal diseases (Schernthaner and Schernthaner, 2013), colorectal cancer (Chen M. et al., 2019), diabetes (Senthilkumar et al., 2017), and pancreatic cancer (Karlsson et al., 2013) possible. However, GLPs can ultimately achieve the reconstruction of microbiota balance and restoration of metabolic homeostasis by reshaping the interaction network of microbiota in these four habitats, regulating the exchange of metabolites and the transmission of signaling molecules among microorganisms, and mediating niche competition (Wu R.-T. et al., 2022; Yang et al., 2019).

In conclusion, GLPs significantly improve insulin resistance, reduce inflammation, and lower blood glucose levels by fostering helpful bacteria’ proliferation while hindering detrimental microorganisms, and reshaping the balance of the microbiota and metabolic homeostasis, thus opening up new avenues for studying the mechanisms of blood glucose reduction (Al-Ishaq et al., 2023; Bielka et al., 2022; Di Vincenzo et al., 2023; Fu et al., 2023; Longo et al., 2023).

## 6 Innovations in combination therapy, structure, and dosage forms

Combination therapy has become a key approach in modern medicine to tackle complex diseases and improve treatment success rates, thanks to its advantages such as synergistic effects, reduced drug resistance, alleviated side effects, and multi-target intervention. Patients have experienced a substantial improvement in quality of life.

The research experiment utilized a diabetic rat model. The designed groups were the normal control group, the model control group, the single fructan group, the single Ganoderma lucidum polysaccharide group, the low-dose fructan + Ganoderma lucidum polysaccharide group (fructan 2.5 g/kg + Ganoderma lucidum polysaccharide 0.2 g/kg), and the high-dose fructan + Ganoderma lucidum polysaccharide group (fructan 5 g/kg + Ganoderma lucidum polysaccharide 0.4 g/kg). The administration period was 5 weeks. Diabetes pathogenesis is intricate and influenced by multiple factors, including blood glucose, lipid profiles, insulin resistance, and oxidative stress. Compared with single-drug use, the combined use of inulin and GLPs notably lowers fasting blood sugar levels while improves the body’s response to insulin, mediates various biological effects of insulin, and improves disorders of conversion of glucose and lipids via regulating various signaling pathways (AMPK pathway, PI3K/Akt pathway, PPAR pathway, and NF-κB pathway) and enhancing body biochemical pathway of utilizes glucose (facilitating relocation of GLUT4 to cell membrane, regulating enzymes related to GK, PFK-1, and GS, and modulating SCFA production); it also improves antioxidant defense capacity by amplifying performance of antioxidant enzymes and reducing levels of ROS. Using drugs in combination provides a multi-target, low-side-effect strategy for the treatment of diabetes and has become a new direction in treatment (Fan et al., 2018; Farhangi et al., 2016; Li et al., 2011; Liu et al., 2019; Mokashi et al., 2017)^.^

Structural modification can optimize drug targeting, reduce toxicity, and improve stability; new dosage forms can achieve controlled release and barrier-crossing delivery, overcoming the bioavailability bottlenec (Cheng et al., 2023; Guo et al., 2020). These two aspects are the core directions of innovation in the current pharmaceutical field. The following is relevant research on the structural modification and development of new dosage forms of GLPs, which aims to provide more effective and safer treatment options and improved prognoses for diabetic patients. The research experiment utilized a diabetic rat model fed with a high-fat diet. A new type of Ganoderma lucidum (Curtis) P. Karst. 1881 (Polyporaceae) polysaccharide-chromium (III) complex [GLP-Cr(III)] has been synthesized in a study. Taking advantage of the biocompatibility and chelating properties of polysaccharides, this complex significantly improves the stability and bioavailability of Cr(III) while mitigating its toxicity. At a reasonable dosage, compared with single GLPs, GLP-Cr(III) significantly reduces fasting blood sugar concentration, TC and TG levels in mice together with shows great potential in preventing and treating hyperglycemia and hyperlipidemia by improving glucose tolerance and regulating the gut microbiota (Li et al., 2019c).

Another study synthesized OGLP-CMC/SA, which exhibits excellent properties including inhibits inflammation, acting against bacteria, and antioxidant effects, besides capability to advance fibroblast proliferation and migration. It demonstrates outstanding therapeutic efficacy in promoting epidermal regeneration and improving treatment of persistent wounds caused by diabetes (Li et al., 2024).

## 7 Conclusion and prospects

Diabetes and its complications have extensive and severe impacts. They can lead to a decline in organ function, a very high disability rate, and a significant shortening of life expectancy, causing a major public health crisis. These conditions can be treated through early intervention, comprehensive management, and patient self-education. GLPs, GLPs, natural metabolites, are sourced from Ganoderma lucidum (Curtis) P. Karst. 1881 (Polyporaceae). It can substantially delay the progression of complications such as kidney disease, liver injury, cardiomyopathy, refractory wound healing, nerve dysfunction, retinopathy, and erectile dysfunction caused by diabetes through multiple mechanisms, including regulating oxidative stress, improving glucose and lipid metabolism disorders, inhibiting cell apoptosis, repairing islet cells, and reducing inflammatory responses. Microbiota dysbiosis is the root cause of many diseases. By reconstructing interdependent connection involving host and its microbiota, multi-dimensional breakthroughs can be achieved in etiological intervention, efficacy improvement, safety enhancement, and cost optimization.

By consulting a large number of literature sources on application of GLPs for diabetes treatment and related complications, the chief mechanism is to ameliorate symptoms through anti-inflammatory, antioxidant, and blocking apoptosis, besides supervision of metabolic processes in organisms. A key finding is that by modulating the gut microbiota, GLPs contribute to the amelioration of diabetes. Microbiota regulation is advancing from empirical approaches to a new stage of precision medicine and is expected to become one of the core strategies for future disease treatment. Current research shows that combination therapy and structural innovation have significant therapeutic effects in treating diabetes and its complications, and will become the core driving force to overcome the bottlenecks in complex disease treatment.

## 8 Limitations and prospects

The application of GLPs in treating diabetes and related complications demonstrates significant promise; however, certain methodological and translational limitations continue to constrain the current body of evidence.

Initially, the differences in extraction techniques, molecular weights, and chemical compositions of GLP samples create considerable heterogeneity, thereby limiting direct comparisons between studies and research on the link between structure and function.125 (Araújo-Rodrigues et al., 2024; Tveden-Nyborg et al., 2024; Wu P. et al., 2024). Secondly, The majority of existing evidence originates from *in vitro* or animal models, lacking adequate clinical validation. This raises questions about its potential effectiveness in treating diabetes in humans (Kim et al., 2025). Third, many of the current investigations suffer from methodological shortcomings, including inadequate control group configurations, failure to assess dose–response relationships, and inconsistent or non-standardized experimental procedures. Fourth, although gut microbiota modulation is considered a primary mode of action for GLPs, it remains uncertain whether observed microbial shifts directly drive metabolic benefits. Lastly, the translation of novel GLP-based formulations—including hydrogel delivery systems and chromium-containing complexes—into clinical practice is hindered by insufficient evidence regarding their safety profile and durability of therapeutic effect (Byndloss et al., 2024).

Future studies should prioritize: (1) establishing standardized protocols and conducting comprehensive physicochemical and biological characterization of GLP preparations; (2) investigating structure–activity relationships to identify and isolate pharmacologically active constituents; (3) implementing rigorously designed, standardized clinical trials to substantiate the efficacy and safety of GLPs in individuals with diabetes. (4) integrating multi-omics approaches with fecal microbiota transplantation (FMT) experiments to elucidate mechanistic links between microbial modulation and therapeutic outcomes; (5) conducting comprehensive assessments of the practical viability and comparative advantages of next-generation GLP formulations and synergistic combination strategies.

Overcoming these limitations is the necessary path to realizing the transition of GLP from basic research to clinical application.

## Data Availability

The original contributions presented in the study are included in the article/supplementary material, further inquiries can be directed to the corresponding authors.

## References

[B1] Agarwal S. Sohal R. S. (1993). Relationship between aging and susceptibility to protein oxidative damage. Biochem. Biophysical Res. Commun. 194, 1203–1206. 10.1006/bbrc.1993.1950 8352777

[B2] Ahagh M. H. Dehghan G. Mehdipour M. Teimuri-Mofrad R. Payami E. Sheibani N. (2019). Synthesis, characterization, anti-proliferative properties and DNA binding of benzochromene derivatives: increased Bax/Bcl-2 ratio and caspase-dependent apoptosis in colorectal cancer cell line. Bioorg. Chem. 93, 103329. 10.1016/j.bioorg.2019.103329 31590040

[B3] Al-Ishaq R. K. Samuel S. M. Büsselberg D. (2023). The influence of gut microbial species on diabetes mellitus. Int. J. Mol. Sci. 24, 8118. 10.3390/ijms24098118 37175825 PMC10179351

[B4] Araújo-Rodrigues H. Sousa A. S. Relvas J. B. Tavaria F. K. Pintado M. (2024). An overview on mushroom polysaccharides: health-Promoting properties, prebiotic and gut microbiota modulation effects and structure-function correlation. Carbohydr. Polym. 333, 121978. 10.1016/j.carbpol.2024.121978 38494231

[B5] Ashriyah R. Wihastuti T. A. Widodo M. A. Sargowo D. (2015). Polisaccharide peptide of Ganoderma lucidum against atherogenesis by reduce foam cells and perivascular adiposite. J. Hypertens. 33, 39. 10.1097/01.hjh.0000469865.84929.2a

[B6] Bi J. Zhou W. Tang Z. (2024). Pathogenesis of diabetic complications: exploring hypoxic niche formation and HIF-1α activation. Biomed. and Pharmacother. 172, 116202. 10.1016/j.biopha 38330707

[B7] Bielka W. Przezak A. Pawlik A. (2022). The role of the gut microbiota in the pathogenesis of diabetes. Int. J. Mol. Sci. 23, 480. 10.3390/ijms23010480 35008906 PMC8745411

[B8] Brem H. Tomic-Canic M. (2007). Cellular and molecular basis of wound healing in diabetes. J. Clin. Investigation 117, 1219–1222. 10.1172/JCI32169 17476353 PMC1857239

[B9] Brownlee M. (2005). The pathobiology of diabetic complications: a unifying mechanism. Diabetes 54, 1615–1625. 10.2337/diabetes.54.6.1615 15919781

[B10] Byndloss M. Devkota S. Duca F. Niess J. H. Nieuwdorp M. Orho-Melander M. (2024). The gut microbiota and diabetes: research, translation, and clinical Applications-2023 diabetes, diabetes care, and diabetologia expert forum. Diabetes 73, 1391–1410. 10.2337/dbi24-0028 38912690 PMC11333376

[B11] Cai Q. Li Y. Pei G. (2017). Polysaccharides from Ganoderma lucidum attenuate microglia-mediated neuroinflammation and modulate microglial phagocytosis and behavioural response. J. Neuroinflammation 14, 63. 10.1186/s12974-017-0839-0 28340576 PMC5364682

[B12] Cao M. Mu X. Jiang C. Yang G. Chen H. Xue W. (2014). Single-nucleotide polymorphisms of GPX1 and MnSOD and susceptibility to bladder cancer: a systematic review and meta-analysis. Tumour Biol. 35, 759–764. 10.1007/s13277-013-1103-6 24037914

[B13] Cefalu W. T. Hu F. B. (2004). Role of chromium in human health and in diabetes. Diabetes Care 36, 2872. 10.2337/diacare.27.11.2741 15505017

[B14] Ceriello A. (2006). Oxidative stress and diabetes-associated complications. Endocr. Pract. 1, 60–62. 10.4158/EP.12.S1.60 16627383

[B15] Chandra M. Chandra N. Agrawal R. Kumar A. Ghatak A. Pandey V. C. (1994). The free radical system in ischemic heart disease. Int. J. Cardiol. 43, 121–125. 10.1016/0167-5273(94)90001-9 8181866

[B16] Chang S.-S. Zhou D. Meng G.-L. Wu F. Wang S. Chen X. (2013). Effect of Ganoderma lucidum polysaccharides on oxidative stress of hyperlipidemic fatty liver in rats. Zhongguo Zhong Yao Za Zhi 37, 3102–3106. 23311162

[B17] Chen C. Chen J. Wang Y. Fang L. Guo C. Sang T. (2022). Ganoderma lucidum polysaccharide inhibits HSC activation and liver fibrosis *via* targeting inflammation, apoptosis, cell cycle, and ECM-receptor interaction mediated by TGF-β/Smad signaling. Phytomedicine 110, 154626. 10.1016/j.phymed.2022.154626 36603342

[B18] Chen Y. Xie M.-Y. Nie S.-P. Li C. Wang Y.-X. (2008). Purification, composition analysis and antioxidant activity of a polysaccharide from the fruiting bodies of Ganoderma atrum. Food Chem. 107, 231–241. 10.1016/j.foodchem.2007.08.021

[B19] Chen L. Liao S. Cheng Q. (2025). Structural insights into polysaccharides in Ganoderma lucidum cell walls by solid-state NMR. Carbohydr. Polym. 367, 123986. 10.1016/j.carbpol.2025.123986 40817531

[B20] Chen M. Xiao D. Liu W. Song Y. Zou B. Li L. (2019). Intake of Ganoderma lucidum polysaccharides reverses the disturbed gut microbiota and metabolism in type 2 diabetic rats. Int. J. Biol. Macromol. 155, 890-902. 10.1016/j.ijbiomac.2019.11.047 31712153

[B21] Chen S. Guan X. Yong T. Gao X. Xiao C. Xie Y. (2022). Structural characterization and hepatoprotective activity of an acidic polysaccharide from Ganoderma lucidum. Food Chem. X, 13, 100204. 10.1016/j.fochx.2022.100204 35499001 PMC9039936

[B22] Chen Y. Zeng X. Gong X. Chen Y. Zhang X. Luo S. (2025). Ganoderma lucidum polysaccharides target the gut-brain axis: unveiling a novel mechanism for ameliorating aging-induced cognitive impairment and oxidative stress. Int. J. Biol. Macromol. 337, 149519. 10.1016/j.ijbiomac.2025.149519 41365407

[B23] Chen Y.-S. Chen Q.-Z. Wang Z.-J. Hua C. (2019). Anti-inflammatory and hepatoprotective effects of Ganoderma lucidum polysaccharides against carbon tetrachloride-induced liver injury in kunming mice. Pharmacology 103, 143–150. 10.1159/000493896 30673679

[B24] Cheng Y. Zhong C. Yan S. Chen C. Gao X. (2023). Structure modification: a successful tool for prodrug design. Future Med. Chem. 15, 379–393. 10.4155/fmc-2022-0309 36946236

[B25] Chiefari E. Arcidiacono B. Foti D. Brunetti A. (2017). Gestational diabetes mellitus: an updated overview. J. Endocrinol. Investigation 40, 899–909. 10.1007/s40618-016-0607-5 28283913

[B26] Chong Z.-L. Zhou M.-C. Chen Y. Xu X.-J. Chen S. Rao J. (2025). Ganoderma lucidum low molecular weight polysaccharide promotes the repair of spinal cord injury through anti-inflammatory and antioxidant. Int. J. Biol. Macromol. 322, 146958. 10.1016/j.ijbiomac 40834957

[B27] Cole J. B. Florez J. C. (2020). Genetics of diabetes mellitus and diabetes complications. Nat. Rev. Nephrol. 16, 377–390. 10.1038/s41581-020-0278-5 32398868 PMC9639302

[B28] Davidson J. A. Scheen A. J. Howlett H. C. S. (2004). Tolerability profile of metformin/glibenclamide combination tablets (Glucovance): a new treatment for the management of type 2 diabetes mellitus. Drug Saf. 27, 1205-1216. 10.2165/00002018-200427150-00004 15588116

[B29] Deng Y. Lei J. Luo X. Wang S. P. Tan H. M. Zhang J. Y. (2025). Prospects of Ganoderma polysaccharides: structural features, structure-function relationships, and quality evaluation. Int. J. Biol. Macromol. 309, 142836. 10.1016/j.ijbiomac.2025.142836 40187470

[B30] Di Vincenzo F. Del Gaudio A. Petito V. Lopetuso L. R. Scaldaferri F. (2023). Gut microbiota, intestinal permeability, and systemic inflammation: a narrative review. Intern. Emerg. Med. 19, 275–293. 10.1007/s11739-023-03374-w 37505311 PMC10954893

[B31] Dong X. Chen Q. Chi W. Qiu Z. Qiu Y. (2024). A metabolomics study of the effects of eleutheroside B on glucose and lipid metabolism in a zebrafish diabetes model. Molecules 29, 1545. 10.3390/molecules29071545 38611823 PMC11013803

[B32] Du Y. Tian L. Wang Y. Li Z. Xu Z. (2024). Chemodiversity, pharmacological activity, and biosynthesis of specialized metabolites from medicinal model fungi Ganoderma lucidum. Chin. Med. 19, 51. 10.1186/s13020-024-00922-0 38519991 PMC10958966

[B33] Duncan B. B. Magliano D. J. Boyko E. J. (2025). IDF diabetes atlas 11th edition 2025: global prevalence and projections for 2050. Nephrol. Dial. Transpl. 41, 7–9. 10.1093/ndt/gfaf177 40874767

[B34] Fadini G. P. Albiero M. Menegazzo L. Boscaro E. Pagnin E. Iori E. (2010). The redox enzyme p66Shc contributes to diabetes and ischemia-induced delay in cutaneous wound healing. Diabetes 59, 2306–2314. 10.2337/db09-1727 20566667 PMC2927954

[B35] Fan Y. He Z. Wang W. Li J. Hu A. Li L. (2018). Tangganjian decoction ameliorates type 2 diabetes mellitus and nonalcoholic fatty liver disease in rats by activating the IRS/PI3K/AKT signaling pathway. Biomed. and Pharmacother. 106, 733–737. 10.1016/j.biopha.2018.06.089 29990865

[B36] Fan X. Liu S. Yu J. Hua J. Feng Y. Wang Z. (2025). Puerarin ameliorates the ferroptosis in diabetic liver injure through the JAK2/STAT3 pathway inhibition based on network pharmacology and experimental validation. Drug Des. Dev. Ther. 19, 737–757. 10.2147/DDDT.S487496 39911447 PMC11796443

[B37] Fang H. Li X. Lin D. Wang L. Yang T. Yang B. (2023a). Inhibition of intrarenal PRR-RAS pathway by Ganoderma lucidum polysaccharide peptides in proteinuric nephropathy. Int. J. Biol. Macromol. 31 (253), 127336. 10.1016/j.ijbiomac.2023.127336 37852403

[B38] Fang H. Lin D. Li X. Wang L. Yang T. (2023b). Therapeutic potential of Ganoderma lucidum polysaccharide peptide in Doxorubicin-induced nephropathy: modulation of renin-angiotensin system and proteinuria. Front. Pharmacol. 14, 1287908. 10.3389/fphar.2023.1287908 37841924 PMC10570435

[B39] Fang Q. Li G. Sun G. Yan H. Li X. Cui S. W. (2025). Protective effect of polysaccharides from Ganoderma atrum against high-fat diet-induced liver injury in mice. Food and Funct. 16 (21), 8484–8495. 10.1039/d5fo03472h 41064915

[B40] Farhangi M. A. Javid A. Z. Dehghan P. (2016). The effect of enriched chicory inulin on liver enzymes, calcium homeostasis and hematological parameters in patients with type 2 diabetes mellitus: a randomized placebo-controlled trial. Prim. Care Diabetes 10 (4), 265–271. 10.1016/j.pcd.2015.10.009 26872721

[B41] Flemming N. B. Gallo L. A. Forbes J. M. (2018). Mitochondrial dysfunction and signaling in diabetic kidney disease: oxidative stress and beyond. Seminars Nephrol. 38 (2), 101–110. 10.1016/j.semnephrol.2018.01.001 29602393

[B42] Flier J. S. Underhill L. H. Brownlee M. Cerami A. Vlassara H. (1988). Advanced glycosylation end products in tissue and the biochemical basis of diabetic complications. N. Engl. J. Med. 318 (20), 1315–1321. 10.1056/NEJM198805193182007 3283558

[B43] Fu Y. Li S. Xiao Y. Liu G. Fang J. (2023). A metabolite perspective on the involvement of the gut microbiota in type 2 diabetes. Int. J. Mol. Sci. 24 (19), 14991. 10.3390/ijms241914991 37834439 PMC10573635

[B44] Gao X. Qi J. Ho C.-T. Li B. Mu J. Zhang Y. (2020). Structural characterization and immunomodulatory activity of a water-soluble polysaccharide from Ganoderma leucocontextum fruiting bodies. Carbohydr. Polym. 249, 116874. 10.1016/j.carbpol.2020.116874 32933694

[B45] Gao M. Zhang W. Ma Y. Liu T. Wang S. Chen S. (2025). Bioactive polysaccharides prevent lipopolysaccharide-induced intestinal inflammation *via* immunomodulation, antioxidant activity, and microbiota regulation. Foods 14 (15), 2575. 10.3390/foods14152575 40807512 PMC12346005

[B46] Gariboldi M. B. Marras E. Ferrario N. Vivona V. Prini P. Vignati F. (2023). Anti-cancer potential of edible/medicinal mushrooms in breast cancer. Int. J. Mol. Sci. 24 (12), 10120. 10.3390/ijms241210120 37373268 PMC10299416

[B47] Genitsaridi I. Salpea P. Salim A. Sajjadi S. F. Tomic D. James S. (2026). 11th edition of the IDF diabetes atlas: global, regional, and national diabetes prevalence estimates for 2024 and projections for 2050. Lancet Diabetes Endocrinol. 14, 149–156. 10.1016/S2213-8587(25)00299-2 41412135

[B48] Giacco F. Brownlee M. (2010). Oxidative stress and diabetic complications. Circ. Res. 107, 1058–1070. 10.1161/CIRCRESAHA.110.223545 21030723 PMC2996922

[B49] Gokce E. C. Kahveci R. Atanur O. M. Gürer B. Aksoy N. Gokce A. (2015). Neuroprotective effects of Ganoderma lucidum polysaccharides against traumatic spinal cord injury in rats. Injury 46 (11), 2146–2155. 10.1016/j.injury.2015.08.017 26298021

[B50] Gopal V. R. Indira M. (2009). Investigations on the correlation of advanced glycated end products (AGE) associated fluorescence with blood glucose and oxidative stress in ethanol-administered diabetic rats. Exp. Toxicol. Pathology. 62 (2), 157–162. 10.1016/j.etp.2009.03.004 19395248

[B51] Graves L. E. Donaghue K. C. (2019). Management of diabetes complications in youth. Ther. Adv. Endocrinol. Metabolism. 10.1177/2042018819863226 31384418 PMC6659178

[B52] Guan Y.-M. Diao Z.-L. Huang H.-D. Zheng J.-F. Zhang Q.-D. Wang L.-Y. (2021). Bioactive peptide apelin rescues acute kidney injury by protecting the function of renal tubular mitochondria. Amino Acids 53 (8), 1229–1240. 10.1007/s00726-021-03028-1 34254213

[B53] Guo L.-M. Sun X.-Z. Liao Y. Li W. (2017). Neuroprotective effects of ganoderma lucidum polysaccharides against oxidative stress-induced neuronal apoptosis. Neural Regen. Res. 12 (6), 953–958. 10.4103/1673-5374.208590 28761429 PMC5514871

[B54] Guo Q. Xu S. Yang P. Wang P. Lu S. Sheng D. (2020). A dual-ligand fusion peptide improves the brain-neuron targeting of nanocarriers in alzheimer's disease mice. J. Control. Release 320, 347–362. 10.1016/j.jconrel.2020.01.039 31978446

[B55] Guo C. Guo D. Fang L. Sang T. Wu J. Guo C. (2021). Ganoderma lucidum polysaccharide modulates gut microbiota and immune cell function to inhibit inflammation and tumorigenesis in Colon. Carbohydr. Polym. 267, 118231. 10.1016/j.carbpol.2021.118231 34119183

[B56] Guo Q. Jin Y. Chen X. Ye X. Shen X. Lin M. (2024). NF-κB in biology and targeted therapy: new insights and translational implications. Signal Transduct. Target. Ther. 9 (1), 53. 10.1038/s41392-024-01757-9 38433280 PMC10910037

[B57] Han H.-S. Kang G. Kim J. S. Choi B. H. Koo S.-H. (2016). Regulation of glucose metabolism from a liver-centric perspective. Exp. and Mol. Med. 48 (3), e218. 10.1038/emm.2015.122 26964834 PMC4892876

[B58] He D. Cui C. (2025). Plant heteropolysaccharides as potential anti-diabetic agents: a review. Curr. Issues Mol. Biol. 47 (7), 533. 10.3390/cimb47070533 40729002 PMC12294070

[B59] He C. Y. Li W. D. Guo S. X. Lin S. Q. Lin Z. B. (2006). Effect of polysaccharides from Ganoderma lucidum on streptozotocin-induced diabetic nephropathy in mice. J. Asian Nat. Prod. Res. 8 (8), 705–711. 10.1080/10286020500289071 17145658

[B60] He J. Z. Shao P. Ni H. D. Chai N. J. Sun P. L. (2010). Study on the structure and constituents of polysaccharide from ganoderma lucidum. Guang Pu Xue Yu Guang Pu Fen Xi 30, 123–127. 20302097

[B61] Hikino H. Ishiyama M. Suzuki Y. Konno C. (1989). Mechanisms of hypoglycemic activity of ganoderan B: a glycan of Ganoderma lucidum fruit bodies. Planta Medica. 55 (5), 423–428. 10.1055/s-2006-962057 2682700

[B62] Hu F. Yan Y. Wang C. W. Liu Y. Wang J. J. Zhou F. (2018). Article effect and mechanism of Ganoderma lucidum polysaccharides on human fibroblasts and skin wound healing in mice. Chin. J. Integr. Med. 25 (3), 203–209. 10.1007/s11655-018-3060-9 30552545

[B63] Hu Y. Wang S.-X. Wu F.-Y. Wu K.-J. Shi R.-P. Qin L.-H. (2022). Effects and mechanism of Ganoderma lucidum polysaccharides in the treatment of diabetic nephropathy in streptozotocin-induced diabetic rats. BioMed Res. Int. 4314415. 10.1155/2022/4314415 35299891 PMC8923773

[B64] Huang C. Kim Y. Caramori M. L. Moore J. H. Rich S. S. Mychaleckyj J. C. (2006). Diabetic nephropathy is associated with gene expression levels of oxidative phosphorylation and related pathways. Diabetes 55 (6), 1826–1831. 10.2337/db05-1438 16731849

[B65] Huang S. Mao J. Ding K. Zhou Y. Zeng X. Yang W. (2017). Polysaccharides from Ganoderma lucidum promote cognitive function and neural progenitor proliferation in mouse model of alzheimer's disease. Stem Cell Rep. 8 (1), 84–94. 10.1016/j.stemcr.2016.12.007 28076758 PMC5233449

[B66] Huang C.-H. Lin W.-K. Chang S. H. Tsai G.-J. (2020). Evaluation of the hypoglycaemic and antioxidant effects of submerged Ganoderma lucidum cultures in type 2 diabetic rats. Mycology 12 (2), 82–93. 10.1080/21501203.2020.1733119 34026300 PMC8128183

[B67] Jia J. Zhang X. Hu Y.-S. Wu Y. Wang Q.-Z. Li N.-N. (2008). Evaluation of *in vivo* antioxidant activities of Ganoderma lucidum polysaccharides in STZ-diabetic rats. Food Chem. 115 (1), 32–36. 10.1016/j.foodchem.2008.11.043

[B68] Jia D. Tang Y. Qin F. Liu B. Hu T. Chen W. (2023). Ganoderma lucidum polysaccharide alleviates Cd toxicity in common carp (cyprinus carpio): neuropeptide, growth performance and lipid accumulation. Comp. Biochem. Physiology C Toxicol. and Pharmacol 271, 109663. 10.1016/j.cbpc.2023.109663 37263520

[B69] Jiang G. Zhang L. Wang H. Chen Q. Wu X. Yan X. (2017). Protective effects of a Ganoderma atrum polysaccharide against acrylamide induced oxidative damage *via* a mitochondria mediated intrinsic apoptotic pathway in IEC-6 cells. Food and Funct. 9 (2), 1133–1143. 10.1039/c7fo01619k 29362765

[B70] Jiang G. Lei A. Chen Y. Yu Q. Xie J. Yang Y. (2021). The protective effects of the Ganoderma atrum polysaccharide against acrylamide-induced inflammation and oxidative damage in rats. Food Funct. 12, 397–407. 10.1039/d0fo01873b 33336655

[B71] Jiang Y. Wang Z. Wang W. Liu Y. Meng Y. Wang Y. (2024). Ganoderma lucidum polysaccharide alleviates cognitive dysfunction by inhibiting neuroinflammation *via* NLRP3/NF-κB signaling pathway. J. Ethnopharmacol. 338 (2), 119065. 10.1016/j.jep.2024.119065 39522844

[B72] Jones J. G. (2016). Hepatic glucose and lipid metabolism. Diabetologia 59 (6), 1098–1103. 10.1007/s00125-016-3940-5 27048250

[B73] Kaneko Y. K. (2016). Development and analysis of novel therapeutic targets to improve pancreatic β-Cell function in type 2 diabetes. Yakugaku Zasshi 136 (12), 1623–1629. 10.1248/yakushi.16-00211 27904096

[B74] Karlsson F. H. Tremaroli V. Nookaew I. Bergström G. Behre C. J. Fagerberg B. (2013). Gut metagenome in European women with normal, impaired and diabetic glucose control. Nature 498 (7452), 99–103. 10.1038/nature12198 23719380

[B75] Kebaili F. F. Tahar N. Esseddik T. M. Redouane R. Chawki B. Pablo A. (2021). Antioxidant activity and phenolic content of extracts of wild Algerian lingzhi or reishi medicinal mushroom, Ganoderma lucidum (agaricomycetes). Int. J. Med. Mushrooms 23 (6), 79–88. 10.1615/IntJMedMushrooms 34369736

[B76] Khosravifar M. Sajadimajd S. Bahrami G. (2023). Anti-diabetic effects of macronutrients *via* modulation of angiogenesis: a comprehensive review on carbohydrates and proteins. Curr. Molecular Medicine 23 (3), 250–265. 10.2174/1566524022666220321125548 35319367

[B77] Khursheed R. Singh S. K. Wadhwa S. Gulati M. Awasthi A. (2020). Therapeutic potential of mushrooms in diabetes mellitus: role of polysaccharides. Int. J. Biol. Macromol. 164, 1194–1205. 10.1016/j.ijbiomac.2020.07.145 32693144

[B78] Kim H. K. Kim S. J. Gil W. J. Yang C. S. (2025). Exploring the therapeutic potential of phytochemicals: challenges and strategies for clinical translation. Phytomedicine 145, 157090. 10.1016/j.phymed.2025.157090 40716124

[B79] Li X. L. Zhou A. G. Li X. M. (2007). Inhibition of Lycium barbarum polysaccharides and Ganoderma lucidum polysaccharides against oxidative injury induced by γ-irradiation in rat liver mitochondria. Carbohydr. Polym. 69 (1), 172–178. 10.1016/j.carbpol.2006.09.021

[B80] Li W.-J. Nie S.-P. Yan Y. Zhu S.-B. Xie M.-Y. (2009). The protective effect of Ganoderma atrum polysaccharide against anoxia/reoxygenation injury in neonatal rat cardiomyocytes. Life Sci. 85 (17-18), 634–641. 10.1016/j.lfs.2009.09.001 19744500

[B81] Li F. Zhang Y. Zhong Z. (2011). Antihyperglycemic effect of Ganoderma lucidum polysaccharides on streptozotocin-induced diabetic mice. Int. J. Mol. Sci. 12 (9), 6135–6145. 10.3390/ijms12096135 22016649 PMC3189773

[B82] Li W.-J. Li L. Zhen W.-Y. Wang L.-F. Pan M. Lv J.-Q. (2016). Ganoderma atrum polysaccharide ameliorates ROS generation and apoptosis in spleen and thymus of immunosuppressed mice. Food Chem. Toxicol. 99, 199–208. 10.1016/j.fct.2016.11.033 27913287

[B83] Li W.-J. Tang X.-F. Shuai X.-X. Jiang C.-J. Liu X. Wang L.-F. (2017). Mannose receptor mediates the immune response to Ganoderma atrum polysaccharides in macrophages. J. Agric. Food Chem. 65 (2), 348–357. 10.1021/acs.jafc.6b04888 27931102

[B84] Li L. Fu W.-W. Wu R.-T. Song Y.-H. Wu W.-Y. Yin S.-H. (2019a). Protective effect of Ganoderma atrum polysaccharides in acute lung injury rats and its metabolomics. Int. J. Biol. Macromol. 142, 693–704. 10.1016/j.ijbiomac.2019.10.010 31739063

[B85] Li L. Li R.-C. Song Y.-H. Wu W.-Y. Yin S.-H. Fu W.-W. (2019b). Effects of a Ganoderma atrum polysaccharide against pancreatic damage in streptozotocin-induced diabetic mice. Food and Funct. 10 (11), 7227–7238. 10.1039/c9fo01990a 31616874

[B86] Li L. Xu J. X. Cao Y. J. Lin Y. C. Guo W. L. Liu J. Y. (2019c). Preparation of Ganoderma lucidum polysaccharide-chromium (III) complex and its hypoglycemic and hypolipidemic activities in high-fat and high-fructose diet-induced pre-diabetic mice. Int. J. Biol. Macromol. 140, 782–793. 10.1016/j.ijbiomac.2019.08.072 31401268

[B87] Li H. N. Zhao L. L. Zhou D. Y. Chen D. Q. (2020). Ganoderma lucidum polysaccharides ameliorates hepatic steatosis and oxidative stress in db/db mice *via* targeting nuclear factor E2 (Erythroid-Derived 2)-Related Factor-2/Heme Oxygenase-1 (HO-1) pathway. Med. Sci. Monit. 26, e921905. 10.12659/MSM.921905 32245940 PMC7154563

[B88] Li T. Zhang Y.-S. Wan M. Wu W. Yao Y.-F. Li W.-J. (2022). Ganoderma atrum polysaccharide modulates the M1/M2 polarization of macrophages linked to the notch signaling pathway. Food and Funct. 13 (7), 4216–4228. 10.1039/d1fo04309a 35332895

[B89] Li F. Liu T. Liu X. Han C. Li L. Zhang Q. (2024). Ganoderma lucidum polysaccharide hydrogel accelerates diabetic wound healing by regulating macrophage polarization. Int. J. Biol. Macromol. 260, 129682. 10.1016/j.ijbiomac.2024.129682 38266851

[B90] Li C. Zhang R. Tian B. Liu B. Mei Y. Li W. (2025). A review of the extraction technologies, structural characterization, chemical modification, and pharmacological effects of Ganoderma lucidum polysaccharides. Naunyn Schmiedeb. Arch. Pharmacol. 398, 16967–16998. 10.1007/s00210-025-04436-w 40719895

[B91] Li F. Liu T. Zhang Q. Wang W. Ma X. Ma R. (2026). Thermosensitive hydrogel with Ganoderma lucidum polysaccharide–ferulic acid nanoparticles and rhEGF for enhanced diabetic wound healing. Chem. Eng. J. 531, 173748. 10.1016/j.cej.2026.173748

[B92] Li H. Xiao Y. Han L. Jia Y. Luo S. Zhang D. (2021). Ganoderma lucidum polysaccharides ameliorated depression-like behaviors in the chronic social defeat stress depression model *via* modulation of Dectin-1 and the innate immune system. Brain Res. Bull. 171, 16–24. 10.1016/j.brainresbull.2021.03.002 33705858

[B93] Li M. Yu L. Zhai Q. Liu B. Zhao J. Zhang H. (2021). Ganoderma applanatum polysaccharides and ethanol extracts promote the recovery of colitis through intestinal barrier protection and gut microbiota modulations. Food and Funct. 13 (2), 688–701. 10.1039/d1fo03677g 34935013

[B94] Li Q. Li H.-J. Xu T. Du H. Huan Gang C.-L. Fan G. (2018). Natural medicines used in the traditional Tibetan medical system for the treatment of liver diseases. Front. Pharmacol. 9, 29. 10.3389/fphar.2018.00029 29441019 PMC5797630

[B95] Li W.-J. Zhang X.-Y. Wu R.-T. Song Y.-H. Xie M.-Y. (2018). Ganoderma atrum polysaccharide improves doxorubicin-induced cardiotoxicity in mice by regulation of apoptotic pathway in mitochondria. Carbohydr. Polym. 202, 581–590. 10.1016/j.carbpol.2018.08.144 30287039

[B96] Liang Z. Yuan Z. Li G. Fu F. Shan Y. (2018). Hypolipidemic, antioxidant, and antiapoptotic effects of polysaccharides extracted from reishi mushroom, Ganoderma lucidum (leysser: fr) karst, in mice fed a high-fat diet. J. Med. Food. 21 (12), 1218–1227. 10.1089/jmf.2018.4182 30183494

[B97] Liang H. Pan Y. Teng Y. Yuan S. Wu X. Yang H. (2020). A proteoglycan extract from Ganoderma lucidum protects pancreatic beta-cells against STZ-induced apoptosis. Biosci. Biotechnol. Biochem. 84 (12), 2491–2498. 10.1080/09168451 32799731

[B98] Liu G. Ji Y. (2024). Electrochemiluminescent evaluation of GLUT4 expression in rat adipocytes induced by Ganoderma lucidum polysaccharides. Int. J. Biol. Macromol. 270 (2), 132106. 10.1016/j.ijbiomac.2024.132106 38734335

[B99] Liu Y. Li Y. Zhang W. Sun M. Zhang Z. (2019). Hypoglycemic effect of inulin combined with ganoderma lucidum polysaccharides in T2DM rats. J. Funct. Foods 55, 381–390. 10.1016/j.jff.2019.02.036

[B100] Liu X. Yang L. Li G. Jiang Y. Zhang G. Ling J. (2022). A novel promising neuroprotective agent: Ganoderma lucidum polysaccharide. Int. J. Biol. Macromol. 229, 168–180. 10.1016/j.ijbiomac.2022.12.276 36587634

[B101] Liu J. Chen Y. Cen Z. Hong M. Zhang B. Luo X. (2025). Ganoderma lucidum spore oil attenuates acute liver injury by modulating lipid metabolism and gut microbiota. J. Pharm. Biomed. Analysis 256, 116674. 10.1016/j.jpba.2025.116674 39842075

[B102] Liu X. Li Y. Wang J. Meng T. Song L. Yang L. (2025). Polysaccharides from Ganoderma lucidum attenuate cognitive impairment in 5xFAD mice by inhibiting oxidative stress and modulating mitochondrial dynamics *via* the Nrf2/antioxidative axis activation. Metab. Brain Dis 40 (4), 180. 10.1007/s11011-025-01601-1 40227285

[B103] Long D. Li Y. Yi S. Lu X. (2025). The protecting role of Ganoderma lucidum polysaccharides on the retinal neurovascular units in rats with retinal ischemia-reperfusion injury. Sci. Rep. 15 (1), 42769. 10.1038/s41598-025-26957-3 41315487 PMC12663397

[B104] Longo S. Rizza S. Federici M. (2023). Microbiota-gut-brain axis: relationships among the vagus nerve, gut microbiota, obesity, and diabetes. Acta Diabetol. 60 (8), 1007–1017. 10.1007/s00592-023-02088-x 37058160 PMC10289935

[B105] Lorenzi M. (1992). Glucose toxicity in the vascular complications of diabetes: the cellular perspective. Diabetes/Metab. Rev. 8 (2), 85–103. 10.1002/dmr.5610080202 1425126

[B106] Lv X.-C. Guo W.-L. Li L. Yu X.-D. Liu B. (2019). Polysaccharide peptides from Ganoderma lucidum ameliorate lipid metabolic disorders and gut microbiota dysbiosis in high-fat diet-fed rats. J. Funct. Foods 57, 48–58. 10.1016/j.jff.2019.03.043

[B107] Ma Q. Zhai R. Xie X. Chen T. Zhang Z. Liu H. (2022). Hypoglycemic effects of Lycium barbarum polysaccharide in type 2 diabetes mellitus mice *via* modulating gut microbiota. Front. Nutr. 9, 916271. 10.3389/fnut.2022.916271 35845787 PMC9280299

[B108] Ma S. Chen Y. Huang H. Pu X. Liang H. Kuang Y. (2025). Structural characteristics, sugar metabolizing enzyme activity and biological activity of Ganoderma lucidum polysaccharides at different growth stages. Sci. Rep. 15 (1), 4834. 10.1038/s41598-025-89559-z 39924541 PMC11808097

[B109] Meng G. Zhu H. Yang S. Wu F. Zheng H. Chen E. (2011). Attenuating effects of Ganoderma lucidum polysaccharides on myocardial collagen cross-linking relates to advanced glycation end product and antioxidant enzymes in high-fat-diet and streptozotocin-induced diabetic rats. Carbohydr. Polym. 84 (1), 180–185. 10.1016/j.carbpol.2010.11.016

[B110] Mfopa A. Mediesse F. K. Mvongo C. Nkoubatchoundjwen S. Lum A. A. Sobngwi E. (2021). Antidyslipidemic potential of water-soluble polysaccharides of Ganoderma applanatum in MACAPOS-2-Induced obese rats. Evidence-based Complementary Altern. Med. 2452057. 10.1155/2021/2452057 34457019 PMC8390130

[B111] Mokashi P. Bhatt L. K. Khanna A. Pandita N. (2017). Swertisin rich fraction from Enicostema littorale ameliorates hyperglycemia and hyperlipidemia in high-fat fed diet and low dose streptozotacin induced type 2 diabetes mellitus in rats. Biomed. and Pharmacother. 96, 1427–1437. 10.1016/j.biopha.2017.09.153 29031588

[B112] Mu X.-Y. Chen S.-B. Yang S.-Y. Wang W.-S. Zhou H.-M. Wang Y.-X. (2025). Ganoderma atrum polysaccharide inhibits ROS/NLRP3/pyroptosis axis by fixing mitochondrial dynamics disorder in PD-1 inhibitors-induced carditis of lewis lung carcinoma mice. Int. J. Biol. Macromol. 310 (2), 143163. 10.1016/j.ijbiomac.2025.143163 40246098

[B113] Pan D. Zhang D. Wu J. Chen C. Xu Z. Yang H. (2013a). Antidiabetic, antihyperlipidemic and antioxidant activities of a novel proteoglycan from ganoderma lucidum fruiting bodies on db/db mice and the possible mechanism. PLOS ONE 8 (7), e68332. 10.1371/journal.pone.0068332 23874589 PMC3708940

[B114] Pan D. Zhang D. Wu J. Chen C. Xu Z. Yang H. (2013b). A novel proteoglycan from Ganoderma lucidum fruiting bodies protects kidney function and ameliorates diabetic nephropathy *via* its antioxidant activity in C57BL/6 db/db mice. Food Chem. Toxicol. 63, 111–118. 10.1016/j.fct.2013.10.046 24211521

[B115] Pan Y. Yuan S. Teng Y. Zhang Z. He Y. Zhang Y. (2022). Antioxidation of a proteoglycan from Ganoderma lucidum protects pancreatic β-cells against oxidative stress-induced apoptosis *in vitro* and *in vivo* . Int. J. Biol. Macromol. 200, 470–486. 10.1016/j.ijbiomac.2022.01.044 35063486

[B116] Pan Y. Zhang Y. Li J. Zhang Z. He Y. Zhao Q. (2023). A proteoglycan isolated from Ganoderma lucidum attenuates diabetic kidney disease by inhibiting oxidative stress-induced renal fibrosis both *in vitro* and *in vivo* . J. Ethnopharmacol. 310, 116405. 10.1016/j.jep.2023.116405 36966849

[B117] Pernicova I. Korbonits M. (2014). Metformin—Mode of action and clinical implications for diabetes and cancer. Nat. Rev. Endocrinol. 10 (3), 143–156. 10.1038/nrendo.2013.256 24393785

[B118] Radziuk J. Pye S. (2001). Hepatic glucose uptake, gluconeogenesis and the regulation of glycogen synthesis. Diabetes/Metabolism Res. Rev. 17 (4), 250–272. 10.1002/dmrr.217 11544610

[B119] Redondo-Horcajo M. Romero N. Martínez-Acedo P. Martínez-Ruiz A. Quijano C. Lourenço C. F. (2010). Cyclosporine A-induced nitration of tyrosine 34 MnSOD in endothelial cells: role of mitochondrial superoxide. Cardiovasc. Res. 87 (2), 356–365. 10.1093/cvr/cvq028 20106845

[B120] Ren B. Zhang Y. F. Liu S. S. Cheng X. J. Yang X. Yang X. G. (2020). Curcumin alleviates oxidative stress and inhibits apoptosis in diabetic cardiomyopathy *via* Sirt1-Foxo1 and PI3K-Akt signalling pathways. J. Cell. Mol. Med. 24 (21), 12355–12367. 10.1111/jcmm.15725 32961025 PMC7687015

[B121] Ren F. Chen Q. Meng C. Chen H. Zhou Y. Zhang H. (2021). Serum metabonomics revealed the mechanism of Ganoderma amboinense polysaccharides in preventing non-alcoholic fatty liver disease (NAFLD) induced by high-fat diet. J. Funct. Foods 82, 104496. 10.1016/j.jff.2021.104496

[B122] Ren L. Zhang J. Zhang T. (2020). Immunomodulatory activities of polysaccharides from ganoderma on immune effector cells. Food Chem. 340, 127933. 10.1016/j.foodchem.2020.127933 32882476

[B123] Ruiz D. F. Z. Chen A. Acosta M. Kim D. V. Callaghan R. C Saldana-Morales F. B. (2023). Microbiota regulation of thymic microbiota-specific T cell development. J. Immunol 210, 218. 10.4049/jimmunol.210.supp.218.15

[B124] Sang T. Guo C. Guo D. Wu J. Wang Y. Wang Y. (2021). Suppression of obesity and inflammation by polysaccharide from sporoderm-broken spore of Ganoderma lucidum *via* gut microbiota regulation. Carbohydr. Polym. 256, 117594. 10.1016/j.carbpol.2020.117594 33483079

[B125] Schernthaner G. Schernthaner G. H. (2013). Diabetic nephropathy: new approaches for improving glycemic control and reducing risk. J. Nephrol. 26 (6), 975–985. 10.5301/jn.5000281 23807645

[B126] Senthilkumar G. P. Anithalekshmi M. S. Yasir M. Parameswaran S. Packirisamy R. Bobby Z. (2017). Role of omentin 1 and IL-6 in type 2 diabetes mellitus patients with diabetic nephropathy. Diabetes and Metabolic Syndrome Clin. Res. and Rev. 12 (1), 23–26. 10.1016/j.dsx.2017.08.005 28864059

[B127] Seweryn E. Ziała A. Gamian A. (2021). Health-promoting of polysaccharides extracted from Ganoderma lucidum. Nutrients 13 (8), 2725. 10.3390/nu13082725 34444885 PMC8400705

[B128] Shahid A. Chen M. Yeung S. Parsa C. Orlando R. Huang Y. (2023). The medicinal mushroom Ganoderma lucidum prevents lung tumorigenesis induced by tobacco smoke carcinogens. Front. Pharmacol. 14, 1244150. 10.3389/fphar.2023.1244150 37745066 PMC10516555

[B129] Shao W. Xiao C. Yong T. Zhang Y. Hu H. Xie T. (2021). A polysaccharide isolated from Ganoderma lucidum ameliorates hyperglycemia through modulating gut microbiota in type 2 diabetic mice. Int. J. Biol. Macromol. 197, 23–38. 10.1016/j.ijbiomac.2021.12.034 34920067

[B130] Shaw J. E. Sicree R. A. Zimmet P. Z. (2009). Global estimates of the prevalence of diabetes for 2010 and 2030. Diabetes Res. Clin. Pract. 87 (1), 4–14. 10.1016/j.diabres.2009.10.007 19896746

[B131] Shi W.-L. Han H. Chen G.-Z. Chen X. Hong Y.-K. Chen L.-K. (2010). Extraction, characterization of the polysaccharide extracts from Se-enriched G. lucidum (Se-GLP) and its inhibition against oxidative damage in ischemic reperfusion mice. Carbohydr. Polym. 80, 774–778. 10.1016/j.carbpol.2009.12.027

[B132] Shi X. Cheng W. Wang Q. Zhang J. Wang C. Li M. (2021). Exploring the protective and reparative mechanisms of G. lucidum polysaccharides against H2O2-Induced oxidative stress in human skin fibroblasts. Clin. Cosmet. Investigational Dermatology 14, 1481–1496. 10.2147/CCID.S334527 34703264 PMC8525518

[B133] Sims E. K. Carr A. L. J. Oram R. A. DiMeglio L. A. Evans-Molina C. (2021). 100 years of insulin: celebrating the past, present and future of diabetes therapy. Nat. Med. 27 (7), 1154–1164. 10.1038/s41591-021-01418-2 34267380 PMC8802620

[B134] Song Q. Liu J. Dong L. Wang X. Zhang X. (2021). Novel advances in inhibiting advanced glycation end product formation using natural compounds. Biomed. and Pharmacother. 140, 111750. 10.1016/j.biopha.2021.111750 34051615

[B135] Sun L.-X. Chen L.-H. Lin Z.-B. Qin Y. Zhang J.-Q. Yang J. (2011). Effects of Ganoderma lucidum polysaccharides on IEC-6 cell proliferation, migration and morphology of differentiation benefiting intestinal epithelium healing *in vitro* . J. Pharm. Pharmacol. 63 (12), 1595–1603. 10.1111/j.2042-7158.2011.01367.x 22060291

[B136] Sun G.-D. Li C.-Y. Cui W.-P. Guo Q.-Y. Dong C.-Q. Zou H.-B. (2015). Review of herbal traditional Chinese medicine for the treatment of diabetic nephropathy. J. Diabetes Res. 2016, 5749857. 10.1155/2016/5749857 26649322 PMC4662991

[B137] Tie L. Li X.-J. Wang X. Channon K. M. Chen A. F. (2009). Endothelium-specific GTP cyclohydrolase I overexpression accelerates refractory wound healing by suppressing oxidative stress in diabetes. Am. J. Physiology-Endocrinology Metabolism 296 (6), E1423–E1429. 10.1152/ajpendo.00150.2009 19336662 PMC2692395

[B138] Tie L. Yang H. Q. An Y. Liu S. Q. Liu J. Xu Y. (2012). Ganoderma lucidum polysaccharide accelerates refractory wound healing by inhibition of mitochondrial oxidative stress in type 1 diabetes. Cell. Physiology Biochem. 29 (3-4), 583–594. 10.1159/000338512 22508065

[B139] Tong A. J. Hu R. K. Wu L. X. Lv X. C. Li X. Zhao L. N. (2019). Ganoderma polysaccharide and chitosan synergistically ameliorate lipid metabolic disorders and modulate gut microbiota composition in high fat diet-fed golden hamsters. J. Food Biochem. 44 (1), e13109. 10.1111/jfbc.13109 31793675

[B140] Tveden-Nyborg P. Yang B. Simonsen U. Lykkesfeldt J. (2024). BCPT perspectives on studies involving natural products, traditional Chinese medicine and systems pharmacology. Basic Clin. Pharmacol. Toxicol. 135, 782–785. 10.1111/bcpt.14109 39617689

[B141] Ubaidillah N. Sargowo D. Widya A. Jakfar V. Proboretno K. S. Failasufi M. (2016). OS 10-03 the distinctive effect of polysaccharide peptides ganoderma lucidum as anti atherogenesis in stable angina patients. J. Hypertens 34, 72. 10.1097/01.hjh.0000500039.08261.4f

[B142] Wan F. Ma F. Wu J. Qiao X. Chen M. Li W. (2022). Effect of Lycium barbarum polysaccharide on decreasing serum amyloid A3 expression through inhibiting NF-κB activation in a mouse model of diabetic nephropathy. Anal. Cell Pathol. (Amst) 2022, 7847135. 10.1155/2022/7847135 35132370 PMC8817866

[B143] Wang S.-Q. Li X. J. Qiu H. B. Jiang Z. M. Simon M. Ma X. R. (2014). Anti-epileptic effect of Ganoderma lucidum polysaccharides by inhibition of intracellular calcium accumulation and stimulation of expression of CaMKII α in epileptic hippocampal neurons. PLOS ONE 9 (7), e102161. 10.1371/journal.pone.0102161 25010576 PMC4092074

[B144] Wang X. Ge Q. M. Bian F. Dong Y. Huang C. M. (2016). Inhibition of TLR4 protects rat islets against lipopolysaccharide-induced dysfunction. Mol. Med. Rep. 32 (4), 257. 10.3892/mmr.2025.13622 28101570

[B145] Wang C. Shi S. Chen Q. Lin S. Wang R. Wang S. (2018). Antitumor and immunomodulatory activities of Ganoderma lucidum polysaccharides in glioma-bearing rats. Integr. Cancer Ther. 17 (3), 674–683. 10.1177/1534735418762537 29607690 PMC6142075

[B146] Wang Y. Yu F. Zheng X. Li J. Zhang Z. Zhang Q. (2023). Balancing adipocyte production and lipid metabolism to treat obesity-induced diabetes with a novel proteoglycan from Ganoderma lucidum. Lipids Health Dis. 22 (1), 120. 10.1186/s12944-023-01880-6 37553709 PMC10408226

[B147] Wang J.-H. Mu X.-Y. Wang W.-S. Li Y.-J. Gui Y.-Q. Peng X.-P. (2025). Ganoderma atrum polysaccharide interferes with TLR4 against PD-1 inhibitors-induced carditis *via* NF-κB-NLRP3 pathway driven by IRF1/VEGFA/14–3-3γ axis in lewis lung carcinoma mice. J. Funct. Foods 127, 106732. 10.1016/j.jff.2025.106732

[B148] Wang L. Zhou Z. Xiang Z. Zhao Y. Li D. Wang Z. (2025). Ganoderma atrum polysaccharides attenuates cadmium mediated hepatorenal dysfunction, oxidative stress, inflammation response, and metabolic disorders in mice. Int. J. Biol. Macromol. 318 (1), 144908. 10.1016/j.ijbiomac.2025.144908 40473162

[B149] Wang Y. Zuo Y. Weng J. Peng X. (2025). Health benefits of Ganoderma lucidum polysaccharides: a review of potential cardiovascular protective effects. Int. J. Biol. Macromol. 330 (1), 148001. 10.1016/j.ijbiomac.2025.148001 41033521

[B150] Wicaksono G. Triharanto A. Ayu I. D. Dewi S. C. Bahri S. Sargowo P. D. (2016). LBPS 01-04 sub chronic toxicity evaluation of polysaccharide peptide ganoderma lucidum in aorta. J. Hypertens 34, 174–175. 10.1097/01.hjh.0000500374.59205.9e

[B151] Wu S. (2018). Hypolipidaemic and anti-lipidperoxidant activities of Ganoderma lucidum polysaccharide. Int. J. Biol. Macromol. 118, 2001–2005. 10.1016/j.ijbiomac.2018.07.082 30009904

[B152] Wu Z.-W. Liu X.-C. Quan C.-X. Tao X.-Y. Yi-Luo N. Zhao X.-F. (2025). Novel galactose-rich polysaccharide from ganoderma lucidum: structural characterization and immunomodulatory activities. Carbohydr. Polym. 362, 123695. 10.1016/j.carbpol.2025.123695 40409828

[B153] Wu G. Liu S. Wang Z. Wang X. (2024). Structural characteristics of neutral polysaccharides purified from coix seed and its anti-insulin resistance effects on HepG2 cells. Food Sci. Nutr. 12, 8419–8431. 10.1002/fsn3.4402 39479660 PMC11521644

[B154] Wu P. Zhang C. Yin Y. Zhang X. Li Q. Yuan L. (2024). Bioactivities and industrial standardization status of ganoderma lucidum: a comprehensive review. Heliyon 10, e36987. 10.1016/j.heliyon.2024.e36987 39435114 PMC11492437

[B155] Wu R.-T. Wang L.-F. Yao Y.-F. Sang T. Wu Q.-L. Fu W.-W. (2022). Activity fingerprinting of polysaccharides on oral, gut, pancreas and lung microbiota in diabetic rats. Biomed. and Pharmacother. 155, 113681. 10.1016/j.biopha.2022.113681 36108392

[B156] Wu T. Cai M. Hu H. Jiao C. Zhang Z. Liu Y. (2022). Whole-genome sequencing and transcriptome analysis of Ganoderma lucidum strain Yw-1-5 provides new insights into the enhanced effect of Tween80 on exopolysaccharide production. J. Fungi. 8 (10), 1081. 10.3390/jof8101081 36294646 PMC9605614

[B157] Xia Q.-H. Lu C.-T. Tong M.-Q. Yue M. Chen R. Zhuge D.-L. (2021). Ganoderma lucidum polysaccharides enhance the abscopal effect of photothermal therapy in hepatoma-bearing mice through immunomodulatory, anti-proliferative, pro-apoptotic and anti-angiogenic. Front. Pharmacol. 12, 648708. 10.3389/fphar.2021.648708 34295244 PMC8290260

[B158] Xiao C. Wu Q.-P. Cai W. Tan J.-B. Yang X.-B. Zhang J.-M. (2012). Hypoglycemic effects of Ganoderma lucidum polysaccharides in type 2 diabetic mice. Archives Pharmacal Res. 35 (10), 1793–1801. 10.1007/s12272-012-1012-z 23139131

[B159] Xiao H. Wu C. Li P. Gao W. Zhang W. Zhang W. (2017). Ratiometric photoacoustic imaging of endoplasmic reticulum polarity in injured liver tissues of diabetic mice. Chem. Sci. 8 (10), 7025–7030. 10.1039/c7sc02330h 29147529 PMC5642195

[B160] Xiao C. Wu Q. Xie Y. Tan J. Ding Y. Bai L. (2018). Hypoglycemic mechanisms of Ganoderma lucidum polysaccharides F31 in db/db mice *via* RNA-seq and iTRAQ. Food and Funct. 9 (12), 6495–6507. 10.1039/c8fo01656a 30467564

[B161] Xie F. Wu M. Lai B. Halim M. Liu S. Shi D. (2020). Effects of redox interference on the pancreatic mitochondria and the abnormal blood glucose. Free Radic. Res. 55 (2), 119–130. 10.1080/10715762.2020.1866180 33327807

[B162] Xu S. Dou Y. Ye B. Wu Q. Wang Y. Hu M. (2017). Ganoderma lucidum polysaccharides improve insulin sensitivity by regulating inflammatory cytokines and gut microbiota composition in mice. J. Funct. Foods 38, 545–552. 10.1016/j.jff.2017.09.032

[B163] Xu Z.-H. Su X. Yang G. Qin T. Liu Y. (2021). Ganoderma lucidum polysaccharides protect against sepsis-induced cardiac dysfunction by activating SIRT1. J. Pharm. Pharmacol. 74 (1), 124–130. 10.1093/jpp/rgab142 34559876

[B164] Yahya M. J. Ismail P. B. Nordin N. B. Akim A. B. M. Yusuf W. Adam N. L. B. (2019). Association of CCL2, CCR5, ELMO1, and IL8 polymorphism with diabetic nephropathy in Malaysian type 2 diabetic patients. Int. J. Chronic Dis. 2019, 2053015. 10.1155/2019/2053015 30713847 PMC6333004

[B165] Yamada Y. Takano Y. Satrialdi N. Abe J. Hibino M. Harashima H. (2020). Therapeutic strategies for regulating mitochondrial oxidative stress. Biomolecules 10. 10.3390/biom10010083 31948035 PMC7023101

[B166] Yang Q. Wang S. Xie Y. Sun J. Wang J. (2009). HPLC analysis of Ganoderma lucidum polysaccharides and its effect on antioxidant enzymes activity and bax, Bcl-2 expression. Int. J. Biol. Macromol. 46 (2), 167–172. 10.1016/j.ijbiomac.2009.11.002 19941892

[B167] Yang Z. Chen C. Zhao J. Xu W. He Y. Yang H. (2017). Hypoglycemic mechanism of a novel proteoglycan, extracted from Ganoderma lucidum, in hepatocytes. Eur. J. Pharmacol. 820, 77–85. 10.1016/j.ejphar.2017.12.020 29233661

[B168] Yang H. Xie T. Li D. Du X. Wang T. Li C. (2019). Tim-3 aggravates podocyte injury in diabetic nephropathy by promoting macrophage activation *via* the NF-κB/TNF-α pathway. Mol. Metab. 23, 24–36. 10.1016/j.molmet.2019.02.007 30862474 PMC6479760

[B169] Yang M. Qin X. Liu X. (2025). A review of polysaccharides from ganoderma lucidum: preparation methods, structural characteristics, bioactivities, structure-activity relationships and potential applications. Int. J. Biol. Macromol. 303, 140645. 10.1016/j.ijbiomac.2025.140645 39909264

[B170] Yao X. Yuan Y. Jing T. Ye S. Wang S. Xia D. (2022). Ganoderma lucidum polysaccharide ameliorated diabetes mellitus-induced erectile dysfunction in rats by regulating fibrosis and the NOS/ERK/JNK pathway. Transl. Androl. Urology 11 (7), 982–995. 10.21037/tau-22-428 35958898 PMC9360518

[B171] YouGuo C. ZongJi S. XiaoPing C. (2009). Modulatory effect of Ganoderma lucidum polysaccharides on serum antioxidant enzymes activities in ovarian cancer rats. Carbohydr. Polym. 78, 258–262. 10.1016/j.carbpol.2009.03.030

[B172] Yu C. Fu J. Guo L. Lian L. Yu D. (2020). UPLC-MS-based serum metabolomics reveals protective effect of Ganoderma lucidum polysaccharide on ionizing radiation injury. J. Ethnopharmacol. 258, 112814. 10.1016/j.jep.2020.112814 32251760

[B173] Yu F. Teng Y. Li J. Yang S. Zhang Z. He Y. (2023). Effects of a Ganoderma lucidum proteoglycan on type 2 diabetic rats and the recovery of rat pancreatic islets. ACS Omega 8 (19), 17304–17316. 10.1021/acsomega.3c02200 37214729 PMC10193549

[B174] Yu L. Lin F. Yu Y. Deng X. Shi X. Lu X. (2024). Rehmannia glutinosa polysaccharides enhance intestinal immunity of mice through regulating the microbiota. Int. J. Biol. Macromol. 283 (3), 137878. 10.1016/j.ijbiomac.2024.137878 39571844

[B175] Zhang H. n Lin Z. b (2004). Hypoglycemic effect of Ganoderma lucidum polysaccharides. Acta Pharmacol. Sin. 25 (2), 191–195. 14769208

[B176] Zhang H.-N. He J.-H. Yuan L. Lin Z.-B. (2003). *In vitro* and *in vivo* protective effect of Ganoderma lucidum polysaccharides on alloxan-induced pancreatic islets damage. Life Sci. 73 (18), 2307–2319. 10.1016/s0024-3205(03)00594-0 12941433

[B177] Zhang J. Liu Y.-J. Park H.-S. Xia Y.-M. Kim G.-S. (2012). Antitumor activity of sulfated extracellular polysaccharides of Ganoderma lucidum from the submerged fermentation broth. Carbohydr. Polym. 87, 1539–1544. 10.1016/j.carbpol.2011.09.051

[B178] Zhang Y.-S. Li W.-J. Zhang X.-Y. Yan Y.-X. Nie S.-P. Gong D.-M. (2017). Ganoderma atrum polysaccharide ameliorates anoxia/reoxygenation-mediated oxidative stress and apoptosis in human umbilical vein endothelial cells. Int. J. Biol. Macromol. 98, 398–406. 10.1016/j.ijbiomac.2017.01.071 28108410

[B179] Zhang S. Pang G. Chen C. Qin J. Yu H. Liu Y. (2018). Effective cancer immunotherapy by Ganoderma lucidum polysaccharide-gold nanocomposites through dendritic cell activation and memory T cell response. Carbohydr. Polym. 205, 192–202. 10.1016/j.carbpol.2018.10.028 30446095

[B180] Zhang C. Li Y. Hu Y. Peng Y. Ahmad Z. Li J.-S. (2019). Porous yolk–shell particle engineering *via* nonsolvent-assisted trineedle coaxial electrospraying for burn-related wound healing. ACS Appl. Mater. and Interfaces 11 (8), 7823–7835. 10.1021/acsami.8b22112 30730130

[B181] Zhang X. Wu D. Tian Y. Chen X. Lan J. Wei F. (2022). Ganoderma lucidum polysaccharides ameliorate lipopolysaccharide-induced acute pneumonia *via* inhibiting NRP1-mediated inflammation. Pharm. Biol. 60 (1), 2201–2209. 10.1080/13880209.2022.2142615 36373992 PMC9665083

[B182] Zhang H. Li N. Zhang Y. Xu Y. Lu F. Lin D. (2024). Ganoderma lucidum polysaccharide peptide alleviates cyclophosphamide-induced Male reproductive injury by reducing oxidative stress and apoptosis. Biomedicines 12 (8), 1632. 10.3390/biomedicines12081632 39200097 PMC11351902

[B183] Zhang H. Jia Q. Song P. Li Y. Jiang L. Fu X. (2025). Incidence, prevalence, and burden of type 2 diabetes in China: trend and projection from 1990 to 2050. Chin. Med. J. Engl. 138, 1447–1455. 10.1097/CM9.0000000000003536 40375461 PMC12180826

[B184] Zhang N. Han Z. Zhang R. Liu L. Gao Y. Li J. (2024). Ganoderma lucidum polysaccharides ameliorate acetaminophen-induced acute liver injury by inhibiting oxidative stress and apoptosis along the Nrf2 pathway. Nutrients 16 (12), 1859. 10.3390/nu16121859 38931214 PMC11206445

[B185] Zhang W. Liu W. Leng F. Shen M. Xie J. (2025). Dietary non-starch plant polysaccharides: multi-mechanisms for managing diabetic microvascular complications. Carbohydr. Polym. 368, 124074. 10.1016/j.carbpol.2025.124074 40912791

[B186] Zhang X. T. Yang Y. Ji C. Fu Y. Pu X. Xu G. (2024). Ganoderma lucidum polysaccharides reduce the severity of acute liver injury by improving the diversity and function of the gut microbiota. Heliyon 10, e35559. 10.1016/j.heliyon.2024.e35559 39170507 PMC11336721

[B187] Zhang Y. Li X. Xu S. Li J. Shi L. Wang Z. (2025). The acetylation of Ganoderma applanatum polysaccharides on ameliorating T2DM-induced hepatic and colonic injuries by modulating the Nrf2/keap1-TLR4/NFκB-Bax/Bcl-2 pathways. Int. J. Biol. Macromol. 294, 140055. 10.1016/j.ijbiomac.2025.140055 39828155

[B188] Zhao S. Lei M. Xu H. He H. Suvorov A. Wang J. (2021). The normal cell proliferation and wound healing effect of polysaccharides from Ganoderma amboinense. Food Sci. Hum. Wellness 10, 508–513. 10.1016/j.fshw.2021.04.013

[B189] Zhao L. Hu H. Zhang L. Liu Z. Huang Y. Liu Q. (2024). Inflammation in diabetes complications: molecular mechanisms and therapeutic interventions. MedComm. 5 (4), e516. 10.1002/mco2.516 38617433 PMC11014467

[B190] Zhen C. Wu X. Zhang J. Liu D. Li G. Yan Y. (2023). Ganoderma lucidum polysaccharides attenuates pressure-overload-induced pathological cardiac hypertrophy. Front. Pharmacol. 14, 1127123. 10.3389/fphar.2023.1127123 37033616 PMC10076566

[B191] Zheng P. Tang Z. Xiong J. Wang B. Xu J. Chen L. (2021). RAGE: a potential therapeutic target during FGF1 treatment of diabetes‐mediated liver injury. J. Cell. Mol. Med. 25 (10), 4776–4785. 10.1111/jcmm.16446 33788387 PMC8107085

[B192] Zhou Z.-Y. Tang Y.-P. Xiang J. Wua P. Jin H.-M. Wang Z. (2010). Neuroprotective effects of water-soluble Ganoderma lucidum polysaccharides on cerebral ischemic injury in rats. J. Ethnopharmacol. 131 (1), 154–164. 10.1016/j.jep.2010.06.023 20600765

[B193] Zhu K. Nie S. Li C. Lin S. Xing M. Li W. (2013). A newly identified polysaccharide from Ganoderma atrum attenuates hyperglycemia and hyperlipidemia. Int. J. Biol. Macromol. 57, 142–150. 10.1016/j.ijbiomac.2013.03.009 23500445

[B194] Zhu K.-X. Nie S.-P. Li C. Gong D. Xie M.-Y. (2014). Ganoderma atrum polysaccharide improves aortic relaxation in diabetic rats *via* PI3K/Akt pathway. Carbohydr. Polym. 103, 520–527. 10.1016/j.carbpol.2013.12.080 24528762

[B195] Zhu K.-X. Nie S.-P. Tan L.-H. Li C. Gong D.-M. Xie M.-Y. (2016). A polysaccharide from Ganoderma atrum improves liver function in type 2 diabetic rats *via* antioxidant action and short-chain fatty acids excretion. J. Agric. Food Chem. 64 (9), 1938–1944. 10.1021/acs.jafc.5b06103 26898215

[B196] Zhu G. Liu Y. Luo S. Tang C. Zhao C. Lu X. (2025). Ganoderma lucidum polysaccharide attenuates retinal ischemia-reperfusion injury by regulating microglial M1/M2 polarization, suppressing neuroinflammation and inhibiting JAK2/STAT3 pathway. Biochem. Biophysics Rep. 41, 101926. 10.1016/j.bbrep.2025.101926 39944465 PMC11815960

[B197] Zou Y. Yang Y. Pei J. Sun P. Wang Y. (2025). Ganoderma lucidum polysaccharide/carboxymethyl chitosan hydrogels modulate macrophage polarization for wound healing. Biomacromolecules 26 (4), 2675–2689. 10.1021/acs.biomac.5c00112 40153544

